# Advances in Extracellular Vesicle-Based Surface-Enhanced Raman Spectroscopy for Cancer Diagnosis

**DOI:** 10.3390/bios16090464

**Published:** 2026-08-26

**Authors:** Shuyuan Zhao, Wen Lei, Juan Li, Jingjing Xia

**Affiliations:** 1 Department of Thoracic and Abdominal Radiation Oncology, Cancer Hospital of Xinjiang Medical University, No. 789 Suzhou Road, Urumqi 830011, China; 2 School of Pharmaceutical Sciences and Institute of Materia Medica, Xinjiang University, No. 777, Hua Rui Street, Shui Mo Gou District, Urumqi 830017, China

**Keywords:** extracellular vesicle, SERS, cancer, biomarker

## Abstract

As a noninvasive liquid biopsy approach, extracellular vesicle (EV)-based detection offers significant advantages in reflecting real-time tumor dynamis and overcoming the limitations of conventional tissue biopsy. EVs, nanoscale vesicles secreted by cells, carry diverse biomolecules such as proteins and nucleic acids, playing key roles in tumor progression, metastasis, and immune evasion, and have emerged as promising biomarkers for cancer liquid biopsy. Surface-enhanced Raman spectroscopy (SERS), characterized by high sensitivity, resistance to photobleaching, minimal sample consumption, and multiplexing capability, has shown great potential in EV analysis. This review systematically summarizes current methods for EV isolation, characterization, and storage, with a focus on label-free and label-based SERS detection strategies for early cancer diagnosis, treatment response monitoring, and prognosis evaluation. Furthermore, the integration of SERS with machine learning and deep learning algorithms has substantially improved diagnostic accuracy and cancer subtyping. Despite remaining challenges, such as optimization of SERS substrate performance, intelligent processing of Raman spectral fingerprints, and clinical translation, EV-based SERS technology holds great promise for precision oncology.

## 1. Introduction

The early diagnosis and treatment of cancer are pivotal for enhancing patient survival rates and optimizing prognosis [1]. To date, tissue biopsy remains the most widely used method for cancer diagnosis [2]. However, a single-site biopsy may not fully represent spatial tumor heterogeneity or support repeated monitoring of tumor evolution, and its invasiveness can limit serial sampling [3]. As an emerging detection approach, liquid biopsy enables the detection of tumor markers in bodily fluids—such as blood and urine—through sample collection. It boasts notable advantages, including noninvasiveness, repeatable sampling, and the capacity to reflect real-time tumor dynamics, thereby exhibiting substantial application potential in early cancer screening, therapeutic efficacy assessment, and recurrence surveillance [4].

Extracellular vesicles (EVs) are lipid-bilayer-enclosed particles released by cells. Exosomes are commonly described as an EV subtype formed through the endosomal pathway and released when multivesicular bodies (MVBs; endosomal compartments containing intraluminal vesicles) fuse with the plasma membrane [5,6]. Because endosomal biogenesis is rarely demonstrated directly in clinical isolates, the Minimal Information for Studies of Extracellular Vesicles 2023 (MISEV2023) guidelines recommend operational terms such as small extracellular vesicles (sEVs) when classification is based mainly on particle size or other physical characteristics [7]. EVs occur in blood, urine, saliva, milk, malignant effusions, and other biological fluids and can contain proteins, lipids, metabolites, and nucleic acids, including DNA, messenger RNA (mRNA), microRNA (miRNA), and long non-coding RNA (lncRNA) [5,6,8]. By transferring selected cargo between cells, tumor-associated EVs can participate in angiogenesis, immune modulation, metastatic niche formation, and therapy resistance [9,10,11]. Their molecular composition is influenced by the cell of origin and its physiological state, although biofluid-derived EV preparations also contain vesicles from non-tumor cells and other co-isolated components [7,12].

Currently, EV isolation and analysis methods [12,13,14,15,16,17,18] primarily include transmission electron microscopy (TEM), Western blot (WB), enzyme-linked immunosorbent assay (ELISA), flow cytometry (FC), nanoparticle tracking analysis (NTA), dynamic light scattering (DLS), gene sequencing, etc. These analytical techniques have significantly advanced EV research, yet they are not without limitations: ELISA, for instance, demands large quantities of highly concentrated samples and fails to enable rapid analysis of complex specimens [19]. NTA has a restricted range for measuring EV concentration, typically 10^6^ to 10^9^ particles/mL [20,21]. FC analysis of EVs may be plagued by problems such as weak signal recovery [22]. TEM, polymerase chain reaction (PCR), and next-generation sequencing technologies are associated with drawbacks such as high detection costs, complex operational procedures, and time inefficiency. In response to the increasing requirements for EV analysis, diverse biosensing technologies have emerged, which enable higher detection sensitivity and significantly optimize the characterization accuracy of EVs [15,23,24,25,26,27,28,29,30,31]. Surface-enhanced Raman spectroscopy (SERS) amplifies Raman scattering via localized electromagnetic enhancement near plasmonic nanostructures, enabling sensitive molecular detection. Compared to fluorescence spectroscopy, SERS spectra are less susceptible to photobleaching and photodegradation, which makes them well suited for long-term dynamic monitoring. Raman peaks typically feature narrow bandwidths, allowing multiplexed detection under single-wavelength excitation. SERS analysis requires only minimal sample volumes, offering distinct advantages for protein detection and the analysis of samples with limited volumes [32,33].

**Scheme 1:**
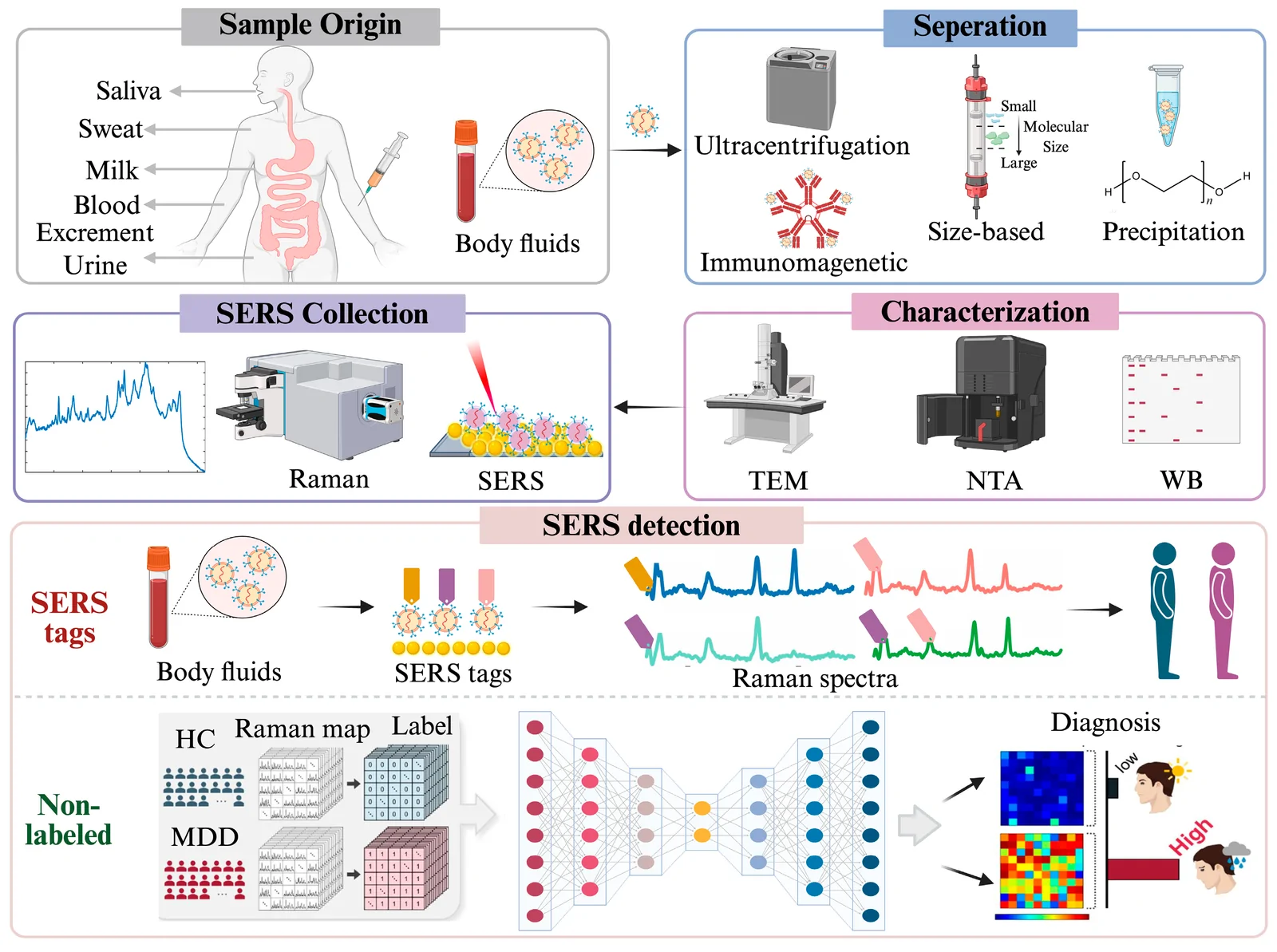
Scheme for the application of EVs combined with SERS in cancer analysis: sample origin → separation (ultracentrifugation, size exclusion, immunomagnetic) → characterization (TEM, NTA, WB) → SERS collection → SERS detection (label-free vs. label-based). The images in the SERS detection section are adapted from Ref. [40]. Created in BioRender. Xia, J. (2026). https://BioRender.com/vf6k4a1.

To develop efficient, minimally invasive liquid biopsy tools, researchers have increasingly investigated SERS-based EV analysis (Scheme 1). The diagnostic and prognostic relevance of EVs arises from several complementary features. EVs can be repeatedly sampled from accessible biofluids, while their lipid bilayer can reduce extracellular degradation of enclosed molecular cargo [34,35]. Tumor-derived EVs may carry molecular features associated with their cells of origin; differences in EV abundance or cargo profiles have therefore been investigated to distinguish patients with cancer from control groups and for tumor classification [11,35]. Because circulating EVs originate from multiple tumor and non-tumor cell populations, their composite profiles may capture aspects of biological heterogeneity, although the cellular origin of most circulating EVs cannot be assigned without selective capture or validated markers [7,11]. In several cancer types, longitudinal changes in selected EV-associated markers have been associated with treatment response or disease progression, and particular signatures have been correlated with TNM stage, metastasis, or survival [35,36]. These findings support the investigation of EVs for diagnosis, treatment monitoring, and prognostic assessment, but they do not yet establish clinical utility; standardized preanalytical procedures and validation in large, prospective, independent cohorts remain necessary [7,35,37]. This potential has also motivated SERS-based EV fingerprinting and computational classification [38,39]. SERS-based EV detection strategies can be broadly categorized as label-free or label-based. Label-free SERS directly records intrinsic Raman fingerprints from the molecular constituents of EV preparations without introducing a Raman reporter. It enables relatively simple and rapid measurements, but overlapping signals from multiple biomolecules and co-isolated components can complicate spectral interpretation. Label-based SERS introduces Raman reporters linked to recognition or encoding systems and identifies or quantifies EV-associated targets through reporter signals. Such formats can improve target-selective detection when validated antibodies, aptamers, or other affinity ligands are used. The Raman label itself does not confer biological specificity. This review summarizes methods for EV isolation, characterization, and storage, discusses label-free and label-based SERS strategies for EV analysis, and evaluates their reported applications in cancer detection, treatment response assessment, and outcome monitoring.

## 2. Extracellular Vesicles

### 2.1. Basic Information on EVs

**Figure 1:**
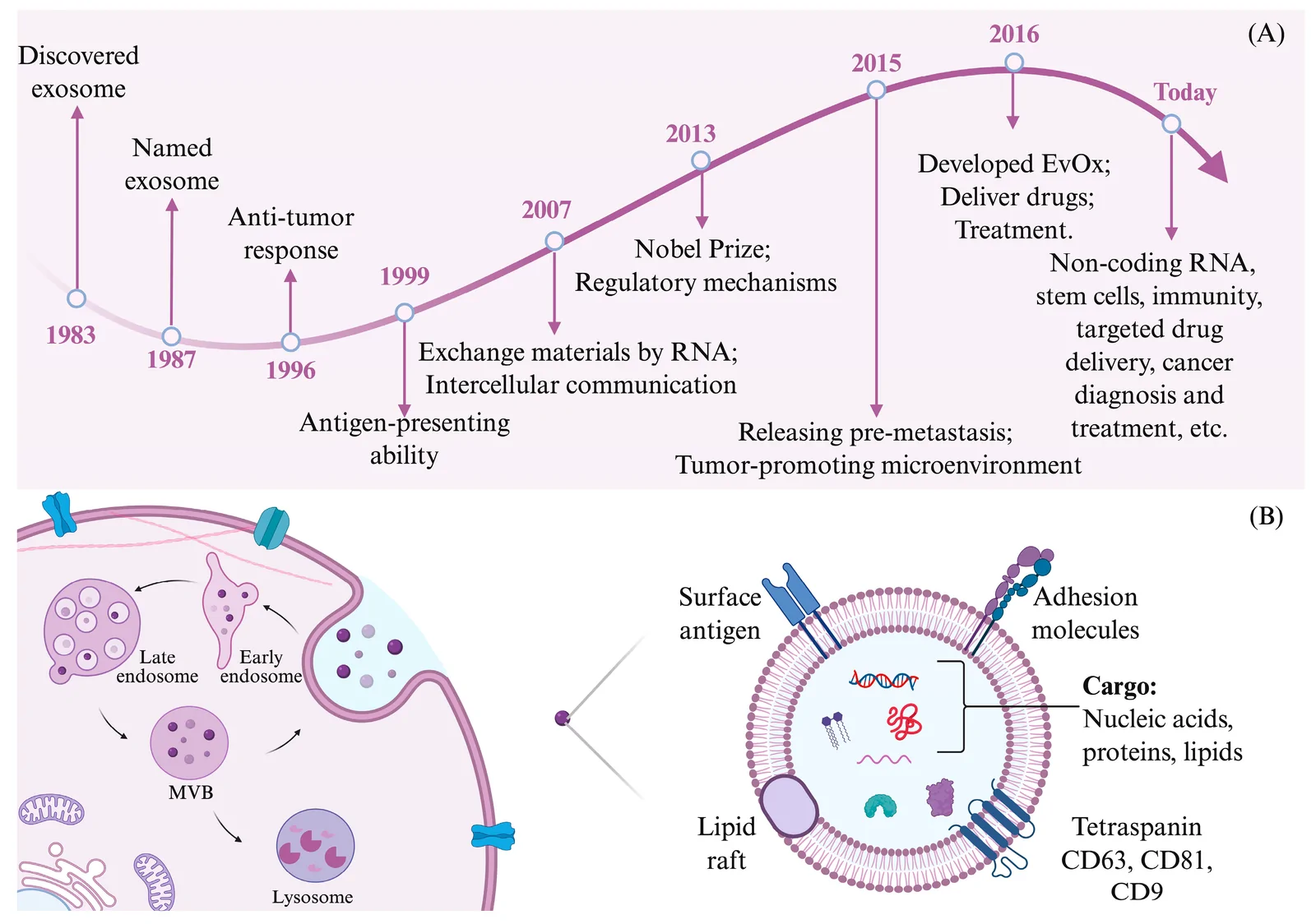
Basic information on EVs. (**A**) A brief history of EVs. (**B**) Endosomal biogenesis and representative cargo of exosomes. Exosomes originate from MVBs and may carry tetraspanins CD63, CD81, and CD9, nucleic acids, proteins, and lipids. Nomenclature follows MISEV2023 operational guidelines. Created in BioRender. Xia, J. (2026). https://BioRender.com/b2n1emx.

Exosomes were first identified in ovine reticulocytes in 1983 [41] and named by Johnstone in 1987 [42]. They are endosome-derived EVs released when multivesicular bodies fuse with the plasma membrane and are generally small, although size alone does not define them [43]. Initially regarded as cellular waste, exosomes were subsequently shown to mediate antigen presentation by B lymphocytes [44] and dendritic cells [45], as well as intercellular transfer of mRNA and miRNAs [46]. These discoveries, together with advances in vesicle transport biology recognized by the 2013 Nobel Prize [47], stimulated research on exosomes in non-coding RNA [48,49], stem cells [50,51], immunity [52,53,54], targeted drug delivery [55], and cancer diagnosis and treatment [56,57,58,59]. Exosomes carry surface antigens, adhesion molecules, nucleic acids, proteins, and lipids within or on a lipid bilayer structure [60]—a molecular complexity that makes them highly amenable to detection by SERS. Specifically, proteins, lipids, and nucleic acids contribute overlapping Raman features, whereas surface antigens govern affinity capture in label-based assays. The developmental history of exosomes is illustrated in Figure 1A.

Exosomes are nanoscale vesicles encapsulated by a phospholipid bilayer and carry a complex molecular cargo. Their cargo includes proteins (such as tetraspanins, adhesion molecules, and cytosolic proteins), lipids, and various nucleic acids, including mRNA, miRNA, lncRNA, and DNA. This structural feature results from their biogenesis, during which they bud inward into multivesicular bodies and are subsequently released upon fusion with the plasma membrane. This feature underpins their role in intercellular communication [8] (Figure 1B).

### 2.2. Methods for EV Isolation

EVs used in SERS studies are isolated from blood, plasma, or serum [33,40,61,62,63,64,65,66,67,68,69,70,71,72], urine [73], and saliva [63], and, when required, fetal bovine serum or bronchoalveolar lavage fluid [74]. Cell-culture-derived EVs are also widely used to distinguish cancerous from non-cancerous sources [61,67,69,75,76,77,78,79,80,81,82,83,84]. Because biogenesis and cargo sorting generate substantial size and molecular heterogeneity [85,86], isolation must balance recovery, purity, structural integrity, and enrichment of the relevant subpopulation [87]. No method provides fully pure EVs or satisfies all requirements for simplicity, speed, throughput, recovery, purity, and cost [88]. Accordingly, researchers should customize EV isolation methods to match their respective research purposes and experimental demands.

#### 2.2.1. Ultracentrifugation-Based Separation

**Figure 2:**
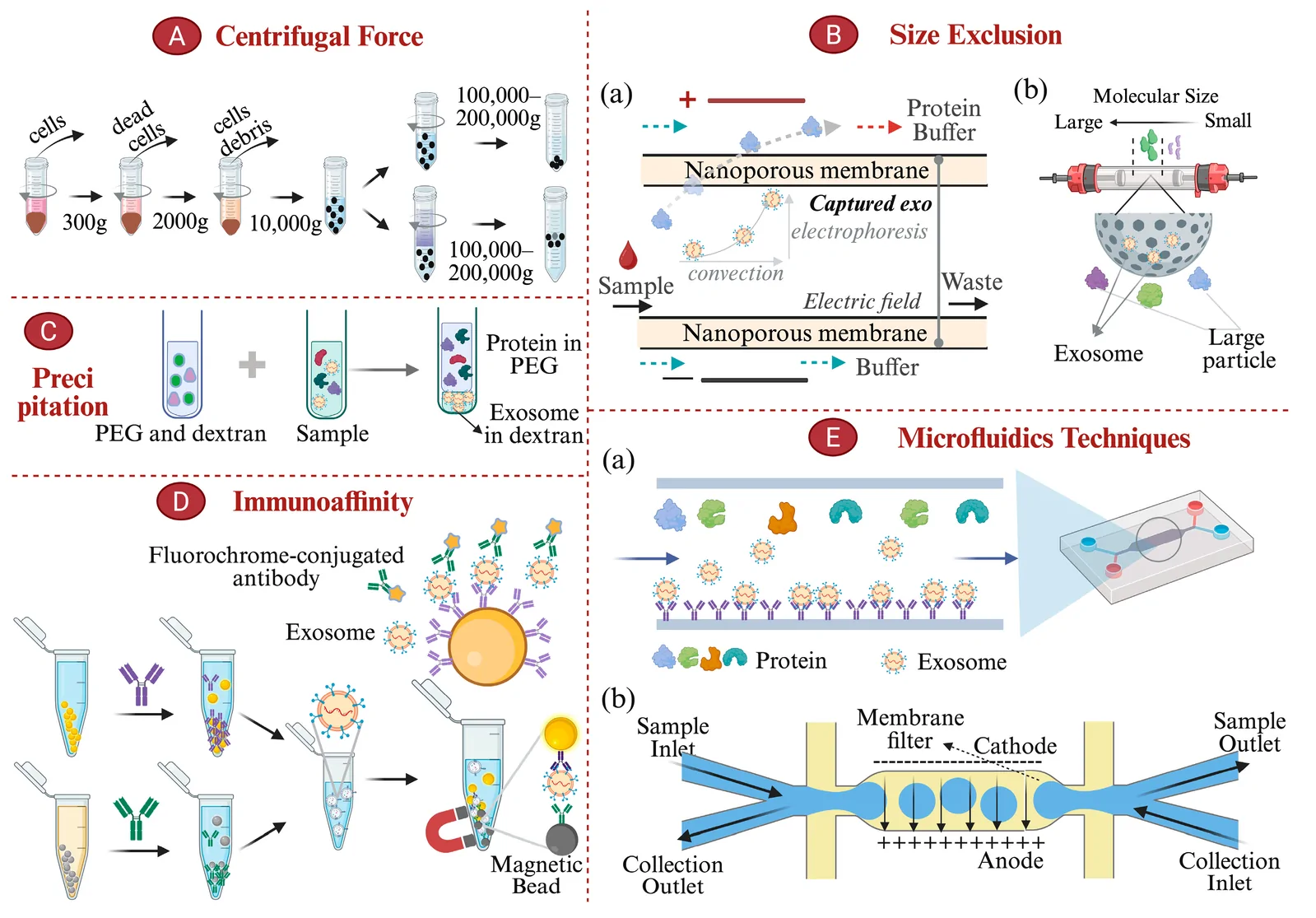
Overview of common EV isolation methods. (**A**) Centrifugal force: sequential spins at 300 g, 2000 g, and 10,000 g remove debris, then ultracentrifugation pellets EVs. (**B**) Size exclusion: (**a**) the nanoporous membrane retains small proteins, while EVs elute; (**b**) the electric field aids separation. (**C**) Precipitation: PEG or PEG/dextran precipitation induces EV aggregation for low-speed collection. (**D**) Immunoaffinity: antibodies on solid supports capture EVs via surface markers. (**E**) Microfluidic: (**a**) antibody-coated microchannels for specific capture; (**b**) membrane filters with electrophoretic assistance for size-based retention. All the above images were created in BioRender. Xia, J. (2026). https://BioRender.com/11lnyjc.

Ultracentrifugation (UC) remains a benchmark and commonly used method [89], which can be categorized into differential UC and density gradient UC. Its working principle stems from the obvious discrepancy in settling velocity between particles of diverse diameters. Big particles sediment quickly, whereas tiny particles need higher centrifugal intensity or extended running time to achieve effective precipitation (Figure 2A). Centrifugal conditions exert remarkable impacts on EV isolation outcomes. Among these key factors, rotor type and centrifugation duration play the most critical roles [90]. Gradient density UC combines UC with gradient media to isolate EVs from nonvesicular particles based on differences in density [91], enabling lower-contamination isolation of EV subpopulations [92] and supporting experiments that require high purity [93]. For SERS, these operating parameters can alter particle concentration, vesicle integrity, and, therefore, the probability of contact with hotspots, while density gradient purification can reduce non-EV spectral background.

Centrifugation is broadly applicable, label-free at the isolation stage, and capable of recovering vesicles across a range of sizes [17]. However, it is time-consuming, requires specialized equipment, and may damage or aggregate vesicles. Viscous samples require more intensive processing. UC can also co-isolate protein aggregates and other vesicles, so additional purification is often required [94]. These effects are particularly important for label-free SERS. Co-isolated biomolecules may be mistaken for EV-associated protein or lipid features. Aggregation and membrane damage can also change local adsorption, hotspot occupancy, and spectral reproducibility.

#### 2.2.2. Size-Based Separation

Size exclusion chromatography (SEC) separates particles by access to porous beads: larger vesicles elute before smaller soluble molecules (Figure 2B) [95]. SEC is simple, removes many soluble proteins, and generally preserves vesicle integrity, but its relatively low yield and sample dilution can limit downstream analysis [96]. To address the dilution issue, SEC is increasingly combined with centrifugal methods [97,98], which concentrate the SEC eluate after size-based removal of cells and large particles [94]. For SERS, the lower protein background improves attribution of label-free fingerprints to vesicles, whereas dilution may weaken signals unless concentration and deposited particle number are controlled.

#### 2.2.3. Polymer Precipitation Techniques

Polymer precipitation techniques primarily utilize polyethylene glycol (PEG) for precipitation. As a hydrophilic agent, PEG competitively binds to water molecules surrounding the EV membrane, reducing EV solubility and promoting aggregation, thereby facilitating precipitation during centrifugation (Figure 2C) [99]. Its performance depends on PEG concentration and molecular mass, ionic strength, pH, and temperature [100]. The method is suitable for large volumes and provides high yields, but co-precipitated proteins and residual polymers reduce purity and may require additional cleanup [95]. PEG-SEC combinations have been used for low-concentration pericardial fluid samples [101] and quantitative proteomic analysis of EV subpopulations [102], and PEG-based principles underpin commercial kits such as Exo-spin and ExoQuick [92]. In SERS, residual PEG and co-precipitated macromolecules can add background, compete for metal surface adsorption, or change aggregation-dependent enhancement; reagent blanks, secondary purification, and matched preparation controls are therefore essential, particularly for label-free fingerprinting [103].

#### 2.2.4. Immunoaffinity Enrichment

Immunoaffinity enrichment is a specific affinity-based isolation strategy that exploits biochemical recognition of EV-associated surface components predominantly through antibodies [104], although alternative recognition elements such as aptamers [105], lipids [106], peptides [107], and glycans [108] have also been developed (Figure 2C). Antibodies are most widely used on magnetic beads or microfluidic surfaces [104] and commonly target CD63, CD9, CD81, CA-125, or EpCAM [109]. In addition, EV isolation can be achieved by lipid-driven hydrophobic interactions with EV membranes rich in amphiphilic phospholipids [106], peptide biomolecules from amino acid condensates [107], or glycan-based recognition of abundant surface O-linked/N-linked glycans, gangliosides and diverse sugar groups [108]. These approaches provide high purity and subgroup specificity but often reduce yield, may modify vesicle surfaces, complicate elution, and remain costly and marker-dependent [110]. For SERS-based applications, affinity capture is well suited to label-based SERS and can improve analytical specificity; however, the signal reflects only marker-positive subpopulations and depends on marker abundance, ligand affinity, and capture efficiency. Accordingly, a low signal cannot be interpreted solely as low total EV abundance, and cross-study comparisons require explicit reporting of the capture target and recovery.

#### 2.2.5. Microfluidic Techniques

Microfluidic lab-on-a-chip platforms provide precise fluid handling at the microscale [111] and allow control of channel geometry, materials, and antibody-functionalized surfaces [112] for particle manipulation [113]. Acoustic, magnetic, or electric fields can further assist separation [114]. These compact devices require small sample volumes, shorten processing time, reduce reagent consumption, and can preserve vesicle morphology. Microfluidic devices achieve capture primarily through immunoaffinity, sieving, or porous trapping [115]. EVs are captured either by binding to specific antibodies immobilized on channel surfaces or by passing through membrane structures (Figure 2(Ea)) [115]. Modified microfluidic systems with surfaces functionalized with biotinylated anti-CD63, control immunoglobulin G (IgG), and anti-CD4 antibodies isolate EVs from 100–400 μL serum and brain tumor specimens, selectively capturing tumor-derived EVs via surface-specific markers to enhance capture; filter membrane-integrated devices purify EVs akin to small vesicle isolation (Figure 2(Eb)) [116]. Microfluidics is especially compatible with integrated SERS. Isolation, concentration, capture, washing, and spectral acquisition can be performed on a single chip, which improves sample utilization and turnaround time. However, channel geometry, surface functionalization, flow rate, nonspecific adsorption, and chip-to-chip variation can alter hotspot exposure and diagnostic output, so device and substrate reproducibility must be evaluated together.

### 2.3. EV Characterization

Obtaining high-quality EV preparations is challenging because co-isolated impurities and structural damage can compromise downstream experiments. Characterization should therefore cover size distribution, concentration, morphology, surface marker expression, and, where possible, non-EV contaminants. For SERS, these measurements are not merely confirmatory: they determine whether spectral differences can reasonably be attributed to vesicle biology rather than unequal particle input, aggregates, contaminants, or processing damage, and they support normalization and between-study comparison.

#### 2.3.1. Visible Characterization

**Figure 3:**
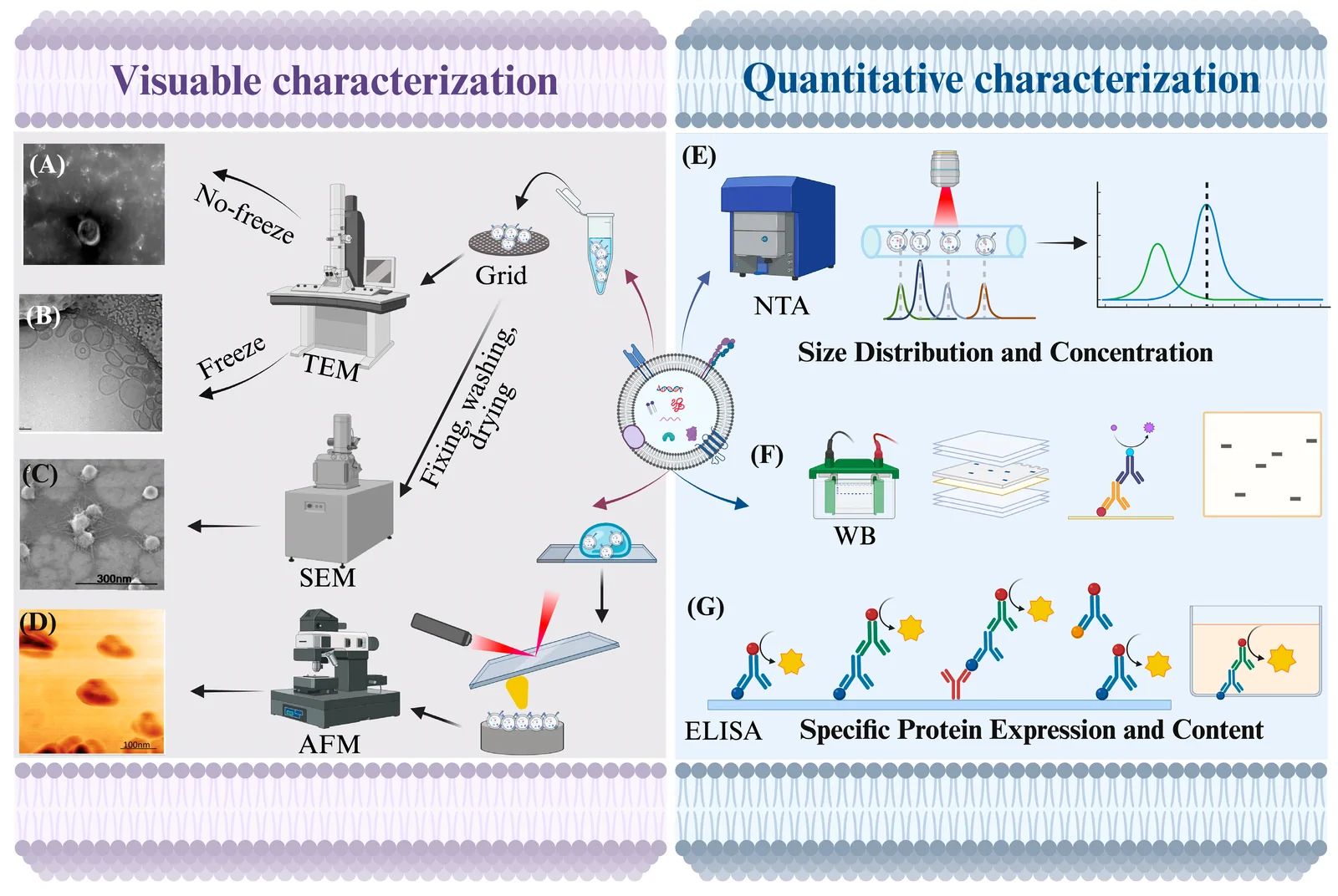
Techniques applied for EV characterization. (**A**) Transmission electron microscopy—cup-shaped morphology [117]. (**B**) Cryo-electron microscopy—native spherical morphology [118]. (**C**) Scanning electron microscopy—surface morphology. Reproduced from Sharma [121]. (**D**) Atomic force microscopy—high-resolution topography. Reproduced from Sharma [121]. (**E**) Nanoparticle tracking analysis—size distribution and concentration. (**F**) Western blot—specific protein expression. (**G**) Enzyme-linked immunosorbent assay—specific protein content. Created in BioRender. Xia, J. (2026). https://BioRender.com/mmcub6a.

Morphological characterization is performed mainly by electron microscopy. Due to variations in operating conditions across different types of microscopes, the observed morphologies of EVs exhibit distinct differences. TEM often shows cup-shaped vesicles (Figure 3A) [117], whereas cryo-electron microscopy avoids fixation and dehydration and more closely preserves spherical native morphology (Figure 3B) [118]. Scanning electron microscopy (SEM) produces surface micrographs (Figure 3C) [119,120] in which saucer-like appearances may reflect collapse during fixation or staining [121]. Atomic force microscopy (AFM) provides surface topography and material property information (Figure 3D) [121], and marker-functionalized tips can recognize and quantify proteins at the single-vesicle level [121,122]. In SERS workflows, morphology helps identify aggregation or membrane disruption that can change vesicle–substrate contact and hotspot occupancy.

#### 2.3.2. Quantitative Characterization

NTA estimates particle size and concentration from light scattering and Brownian motion while analyzing vesicles in suspension (Figure 3E), but particles below approximately 70 nm may be missed, and hydrodynamic diameter may be overestimated [123,124]. DLS measures size distribution and zeta potential from scattered-light fluctuations [124,125,126] but reports an ensemble average and is strongly influenced by protein aggregates or larger vesicles [126].

The MISEV2023 guidelines retain the five marker protein categories established in MISEV2018 and recommend assessing at least three positive markers, including transmembrane and cytosolic proteins, and one negative marker [7]. No universal exosome marker exists; proposed biogenesis-related markers such as Annexin A1, solute carrier family 3 member 2 (SLC3A2), basigin (BSG), and lysosome-associated membrane protein 1 (LAMP1) are not yet universally accepted. Even the most widely used tetraspanins—CD9, CD63, and CD81—are neither exosome-specific nor present on every vesicle. Western blotting (WB), ELISA, and nanoflow cytometry can evaluate marker expression and sample purity: WB provides molecular weight information but is labor-intensive (Figure 3F), ELISA is sensitive and high-throughput but requires suitable epitopes and remains susceptible to antibody cross-reactivity (Figure 3G), and nanoflow cytometry supports multiparameter analysis. These controls are especially important for SERS. In affinity-based assays, marker abundance and heterogeneity directly determine capture and signal in label-free assays; positive and negative markers help establish that the measured spectrum originates predominantly from EVs rather than co-isolated material [7].

The characterization of EVs varied across the SERS studies reviewed here. Although many studies reported particle size, morphology, and one or more tetraspanin markers, they less frequently assessed negative markers and common non-EV contaminants. Many analyzed samples are thus more appropriately described as EV- or sEV-enriched preparations rather than confirmed exosomes. This is especially true for label-free studies, where co-isolated proteins and lipoproteins may contribute to the measured spectra [7,63,83].

### 2.4. Storage of EVs

EVs are vesicular structures enclosed by a double-layered lipid membrane, which functions to protect the structural integrity and biological activity of internal biomolecules. However, the integrity and bioactivity of isolated EVs are susceptible to being impacted by storage temperature and medium [127].

#### 2.4.1. Storage Temperature

**Figure 4:**
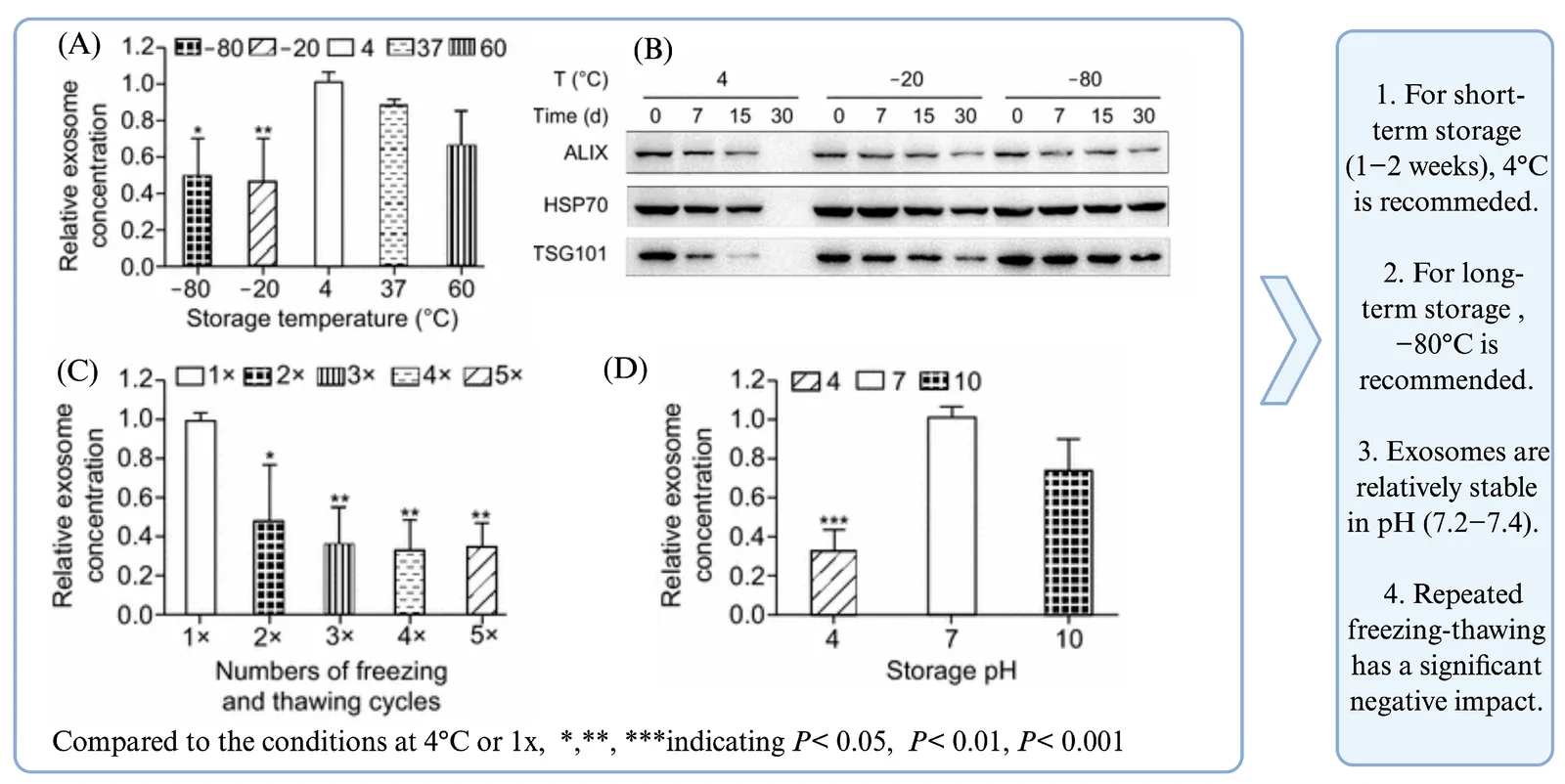
Storage of EVs. (**A**) Changes in relative EV concentration with storage temperature [128]. (**B**) Protein expression of EVs under different storage temperatures and durations [128]. (**C**) Changes in relative EV concentration with the number of freeze–thaw cycles [128]. (**D**) Changes in relative EV concentration with storage pH [128].

Cryopreservation is the most common storage method for EVs, yet different low-temperature conditions differentially affect their morphology, physical properties, and multilamellar vesicle formation. Cheng et al. [128] summarized the effects of storage temperature, pH, and freeze–thaw cycles on EV morphology, particle size, number, cargo, and function, revealing temperature-dependent damage. Short-term storage at 4 °C avoids freeze–thaw membrane damage and optimally maintains protein activity (Figure 4A), but after 1–2 weeks at 4 °C, EVs show increased particle size, degradation of 312 proteins, and loss of biological function (Figure 4B) [128,129]. For longer storage, −80 °C generally preserves function better, although reduced zeta potential and increased particle size indicate aggregation. Repeated freeze–thaw cycles alter cargo and surface markers, and even one cycle may reduce recoverable EV content by approximately 50% (Figure 4C) [130,131,132]. For SERS, aggregation changes hotspot occupancy, whereas protein degradation, membrane disruption, and cargo leakage alter peak intensities and multivariate spectral patterns. Therefore, control samples must be matched for storage time, temperature, and freeze–thaw cycles, and these variables should be recorded in diagnostic studies [133].

#### 2.4.2. Storage Medium

Storage at −80 °C is widely used for long-term EV preservation [134]. Cryoprotectant-assisted vitrification forms an amorphous matrix below the glass transition temperature, limiting ice crystal damage [37], and storage without an appropriate medium can reduce EV counts [135]. Cryoprotectants include permeating agents, such as glycerol, dimethyl sulfoxide (DMSO), and propylene glycol, and non-permeating saccharides, such as trehalose. Glycerol has little effect on recovery, whereas DMSO may improve it [136] by increasing membrane permeability and promoting water efflux before freezing [132]. Trehalose stabilizes proteins, membranes, and liposomes [137]; reported recovery was approximately fourfold higher than in a culture medium, with about 90% remaining after two weeks at 4 °C and 80% after three freeze–thaw cycles. Beyond cryoprotectants, the pH and composition of the storage buffer also influence EV stability. Near-neutral pH better preserves EV content, whereas acidic or alkaline conditions, especially acidic storage, reduce it (Figure 4D). Long-term storage in phosphate-buffered saline can also decrease recovery; accordingly, MISEV2023 does not prescribe a universal storage condition [133]. For SERS, cryoprotectants, residual storage media, buffer choice, and pH may introduce their own Raman bands. They may also alter ionic strength or adsorption onto the plasmonic surface, and they can affect nanoparticle aggregation. Therefore, before spectral comparison, it is necessary to use matched medium blanks and, when compatible with recovery, to remove residual cryoprotectant.

## 3. Raman Information of EVs

The link between cancer and the Raman signature of EVs lies in the altered biomolecular composition of tumor-derived EVs. Oncogenic processes such as abnormal lipid metabolism, differential protein expression, and nucleic acid alterations change the relative abundance and conformation of lipids, proteins, and nucleic acids carried by EVs. These changes produce characteristic variations in SERS peak intensities, ratios, and, occasionally, peak shifts [77]. These spectral variations give rise to distinct spectral characteristics in EVs from different sources and states, which can serve as potential biomarkers for cancer diagnosis [78]. For small molecules, related Raman peaks include thiocyanate, which corresponds to 443 and 553 cm^−1^ [138]. For amino acid residues in proteins: Tyrosine shows characteristic peaks at 638, 654, 660, 830, 835, 1571 and 1614 cm^−1^ [77,80,103,138]. Tryptophan gives Raman peaks at 753, 867, and 1566 cm^−1^ [77,103]. Proline presents peaks at 867, 930, 939, 947, and 1044 cm^−1^ [77,83,103,138]. Phenylalanine corresponds to 630, 999, 1002, 1012, 1111, and 1580 cm^−1^ [77,103,138]. The specific peak positions of the protein are 521, 826, 970, 1049, 1435, 1450, 1542, 1592, 1595, 1618, 1620, and 1632 cm^−1^ [77,78,103,138]. For nucleic acids (DNA, nucleic acid, etc.): DNA has characteristic peaks at 668, 722, 1490, 1510, 1592, and 1664 cm^−1^ [78,83]. Nucleic acids correspond to 786–816, 1100, 1243, 1254, 1323, 1655, and 1662 cm^−1^ [78,138]. Adenine gives 631, 722, 736, 1571, and 1578 cm^−1^ [83,138]. Guanine and cytosine are expressed at 1180, 1183, 1287, 1340, 1360, 1380, 1576, and 1577 cm^−1^ [80,83,138]. For lipids (lipids, phospholipids, etc.), there are numerous lipid-related peaks, like 716, 826, 1066, 1070, 1140, 1179, 1211, 1305, 1307, 1381, 1395, 1447, 1449, 1450, 1455, and 1465 cm^−1^ [78,80,83,138], related to bending, twisting, and stretching of groups (e.g., CH_2_, CH_3_) in lipid molecules and phospholipid characteristic peaks, which are applicable for studying lipid types and membrane structures. Carbohydrates (saccharides, glycoproteins, etc.) have peaks at 593, 835, 930, 939, and 1370 cm^−1^, which assist in analyzing carbohydrate components in biological samples [83,138]. Cholesterol has characteristic peaks at 406, 546, 864, 867, 1435, and 2890 cm^−1^, which are usable for detecting its presence and content [78,80,138].

It should be noted that the peak positions listed in Table 1 are compiled primarily from SERS studies [78,80,83,139]. They should not be regarded as fixed reference spectra that can be directly applied to conventional Raman spectroscopy. SERS can shift peak positions and alter relative intensities because of surface selection rules and hotspot geometry [78,80]. Overlapping bands from proteins, lipids, nucleic acids, and co-isolated contaminants, such as plasma proteins and lipoproteins, may further complicate interpretation [63,83]. Substrate composition and nanogap structure can also selectively enhance different molecular components, leading to substrate-dependent spectral variations [78,80,139]. All peak assignments in the table should be considered tentative and are valid only under the specific sample matrix, preparation protocol, and SERS substrate conditions used. A definitive judgment about a particular molecule or disease state should not be based solely on a single peak position.

**Table 1:** Reported peak positions and tentative band assignments of EVs.

Raman Shift (cm^−1^)	Tentative Band Assignment	Ref.	Raman Shift (cm^−1^)	Tentative Band Assignment	Ref.
406	Cholesterol	[78]	1180	Guanine or cytosine	[83]
443	Thiocyanate	[138]	1183	Adenine, guanine, or cytosine	[83]
460	Saccharide	[138]	1211	Phospholipids	[78]
521	Protein	[78]	1220	Amide III, Tyrosine	[80]
533	Thiocyanate	[138]	1243, 1254	Nucleic acids	[78]
546	Cholesterol	[78]	1286	Fatty acids	[78]
593	Saccharide	[138]	1287	Cytosine	[103]
630	Phenylalanine	[77]	1305	CH_3_ and CH_2_ twisting in lipids	[83]
631	Adenine	[77]	1307	C–N stretching in lipids	[78]
638	Tyrosine	[138]	1323	CH_2_–CH_2_ vibration; nucleic acid-associated band	[138]
654	Tyrosine or guanine	[77]	1340	Guanine (nucleic acid)	[80]
660	C-C twisting of Tyrosine	[80]	1360	Guanine	[83]
668	DNA	[78]	1370	Saccharide	[83]
716	C–N^+^(CH_3_)_3_ vibration of phospholipids	[103]	1380	Guanine	[77]
722	Adenine: ν(C–C) and ν(C–O), DNA	[83]	1381, 1395	CH_3_ symmetric deformation and scissoring in lipids	[78]
736	Adenine	[77]	1435	CH_2_/CH_3_ scissoring in lipids and proteins	[80]
753	Tryptophan	[103]	1438	Cholesterol	[103]
786, 816	Nucleic acids	[78]	1447, 1449	C–H vibration and CH_2_ bending in lipids	[83]
826	Phospholipid C–O–O vibration, protein-associated band	[77]	1450	CH_2_/CH_3_ deformation in lipids and proteins	[83]
830, 835	Stretching, saccharide-associated band at 835 cm^−1^	[80,83]	1455, 1465	Lipids	[80]
864, 867	Cholesterol, proline- or tryptophan-associated band at 867 cm^−1^	[77]	1490, 1510	DNA bases; ring-breathing modes	[78]
930, 939	Proline	[138]	1542	Protein	[78]
947	Proline- or saccharide-associated band	[83]	1566	Tryptophan	[103]
970	Lipid-, protein-, or nucleic acid-associated band	[103]	1571	Tyrosine or adenine	[77]
999	Phenylalanine	[77]	1576, 1577	Guanine	[77]
1002	Phenylalanine C–C symmetric stretching or tryptophan ring-breathing mode	[138]	1578	Adenine, guanine	[83]
1012	Phenylalanine	[103]	1580	Phenylalanine	[77]
1044	Proline-associated band, ν_3_(PO_4_^3−^) symmetric stretching	[103]	1592	Protein- or DNA-associated band	[103]
1049	C–N and C–C stretching of lipids and proteins	[138]	1595	C=O stretching in proteins	[83]
1066, 1070	C–C chain stretching in lipids	[83]	1614	Tyrosine	[103]
1087	Phosphate group vibration	[33]	1618, 1620	C=C stretching, protein-associated band	[78]
1100	Nucleic acids	[138]	1632	Protein	[78]
1111	Phenylalanine/protein-associated band	[103]	1655, 1662	Nucleic acids	[138]
1140	Fatty acids	[103]	1664	DNA	[78]
1145, 1149	Protein; CH_2_ bending	[77]	2890	CH_2_ stretching, cholesterol-associated band	[80]
1179	Phospholipids	[78]			

## 4. EV-Based SERS in Cancer Diagnosis

**Figure 5:**
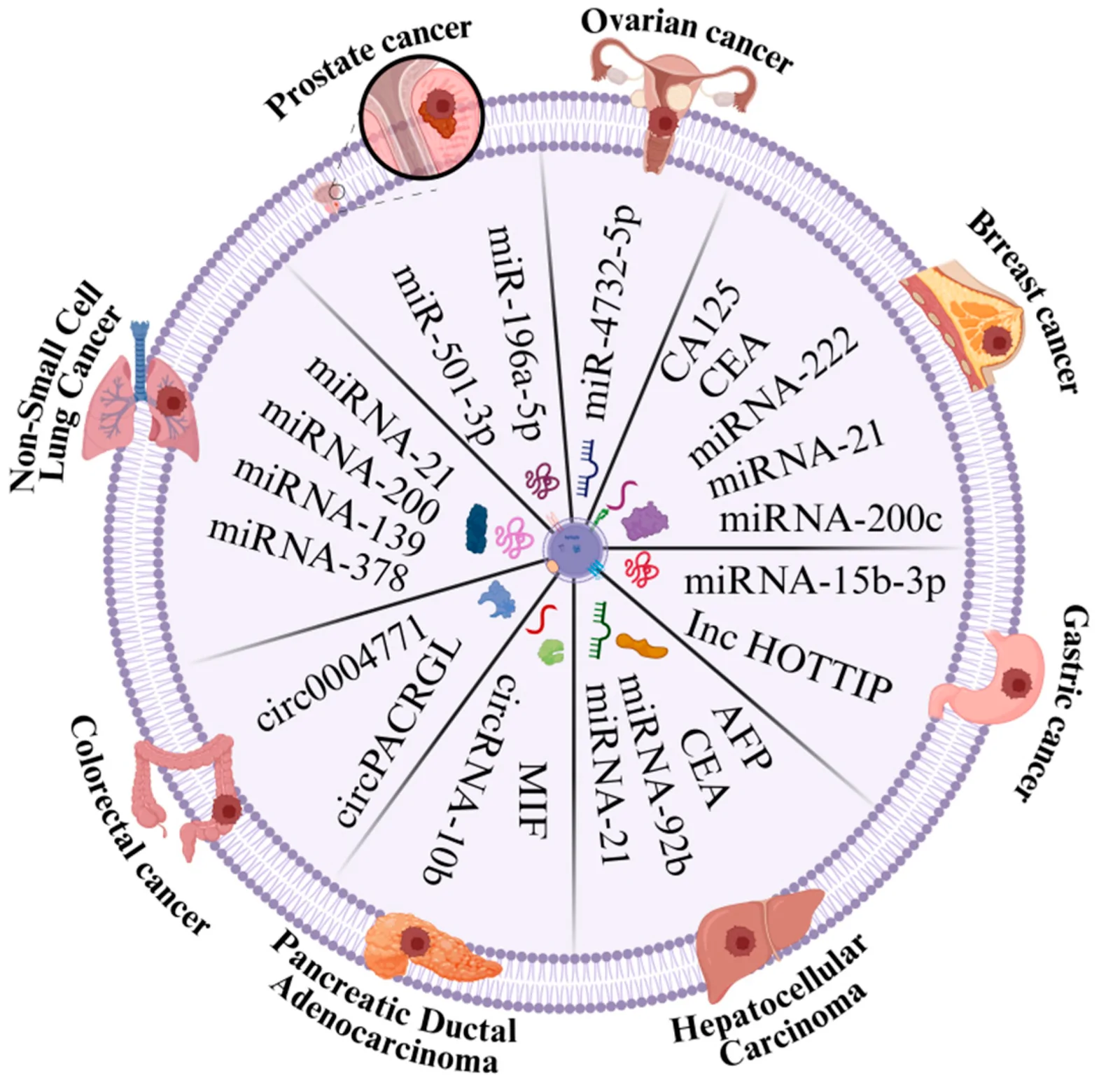
Summary diagram of EV-associated biomarkers for cancer diagnosis and prognosis. The diagram shows EV-associated miRNAs (miRNA-200c, miRNA-15b-3p, miRNA-21), circRNAs (circ0004771, circPACP, circRNA-104), and proteins [Carcinoembryonic Antigen (CEA), Alpha-Fetoprotein (AFP), Macrophage Migration Inhibitory Factor (MIF)] associated with prostate cancer, ovarian cancer, gastric cancer, gastric adenocarcinoma, and hepatocellular carcinoma. Created in BioRender. Xia, J. (2026). https://BioRender.com/doactil.

EVs, emerging as a novel class of cancer biomarkers, exhibit significant potential in facilitating the diagnosis of various malignancies, including lung cancer, breast cancer, gastrointestinal cancers, hepatocellular carcinoma, pancreatic cancer, urinary system cancers, gynecological cancers, hematological malignancies, and melanoma. Figure 5 presents EV-associated biomarkers commonly used in clinical settings for cancer diagnosis and prognostic assessment. Research indicates that tumor cells secrete a greater quantity of EVs than normal cells, and these tumor-derived EVs also contain a range of dysregulated proteins and RNAs, which serve as valuable indicators for cancer diagnosis and prognosis. Accordingly, this section reviews specific label-based and nonspecific label-free SERS detection strategies and then discusses artificial intelligence (AI)-assisted spectral analysis for extracting diagnostic information from complex EV-associated Raman signals.

### 4.1. Label-Based Detection of EVs Using SERS

**Figure 6:**
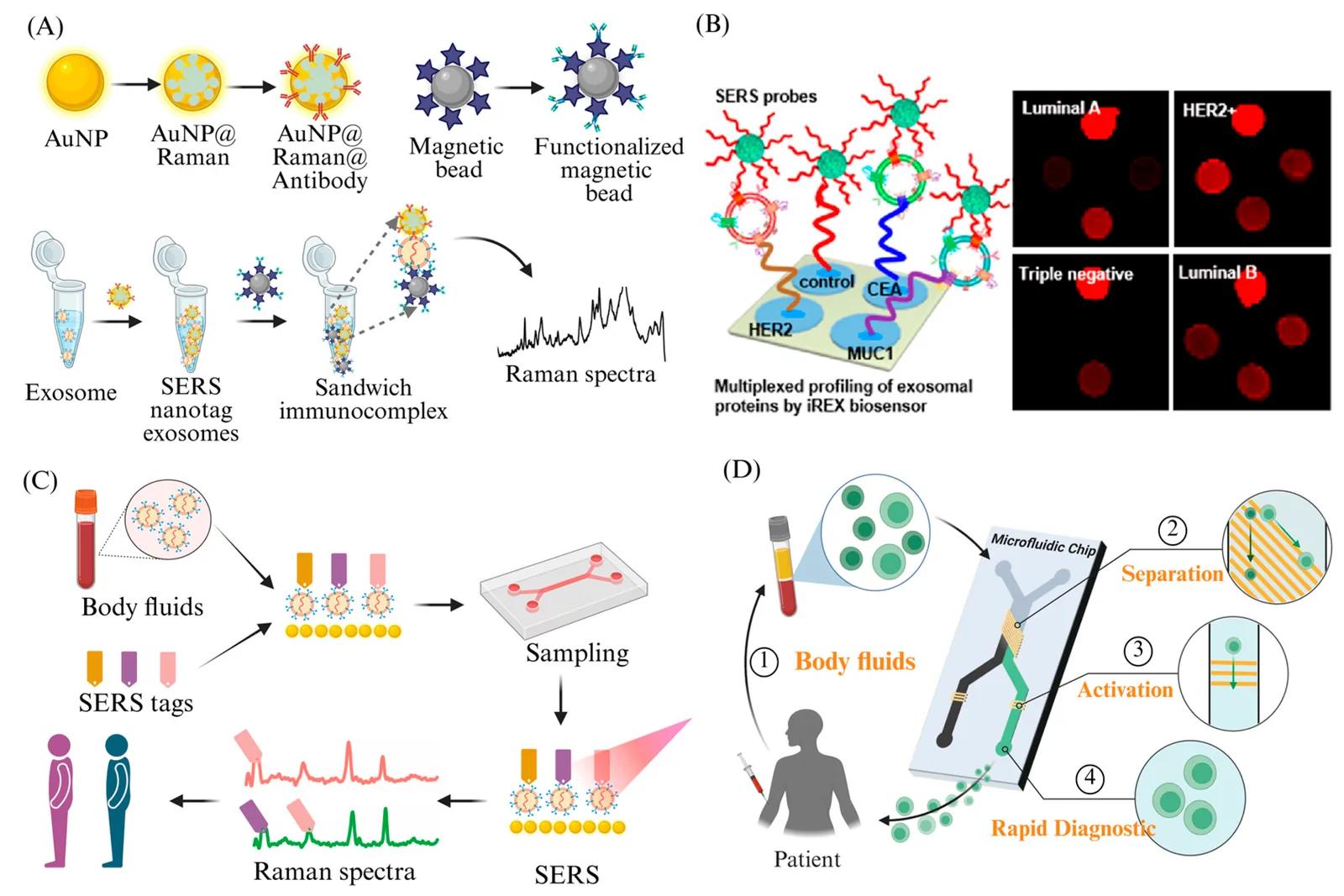
Specific detection of EVs via SERS. (**A**) “Sandwich” SERS immunoassay for EVs. (**B**) Paper-based SERS-vertical flow biosensor for multiplexed quantitative profiling of EV-associated proteins [144]. (**C**) SERS-based profiling of multiple biomarkers in plasma-derived EVs. (**D**) Structure and operational mechanism of a SERS-based on-chip EV analysis platform. Created in BioRender. Xia, J. (2026). https://BioRender.com/e23gczj.

Label-based SERS detection is a technique that combines SERS spectroscopy with specific antibody–antigen interactions. SERS nanotags are a class of plasmonic nanoparticles (NPs) with intense Raman signals. Through specific reactions analogous to antibody–antigen interactions, SERS nanotags bind to target EVs to form a “sandwich” complex. Afterwards, the Raman intensity of Raman reporter molecules in this composite structure is detected to indirectly characterize the distribution and concentration of target analytes. Typical “sandwich” structures generally utilize two types of nanoparticles, namely, magnetic nanobeads and SERS nanoprobes. The surfaces of these two particles are functionalized with two specific antibodies, each of which can specifically bind to two different proteins present on EVs. Target EVs facilitate the formation of sandwich-style immune assemblies consisting of SERS tags, EVs and magnetic nanocarriers. Applying an external magnet enables the enrichment and sedimentation of such immune conjugates, whose SERS signals can be readily measured. In the absence of EV targets, no specific immune binding occurs; the sediment merely contains magnetic nanoparticles and shows nearly no SERS response. Therefore, EV detection can be realized by monitoring SERS signal changes (Figure 6A).

Studies in this category have focused on optimizing sandwich immunoassays and extending them toward active enrichment and multiplexed detection. Numerous fabrication methods for sandwich immunocomplexes have been developed, yet few works have compared their detection performance. A recent study evaluated three diverse technical routes for EV detection. It was found that the highest signal intensity was obtained by incubating small EVs with SERS nanoprobes before testing, achieving a limit of detection (LOD) of 1.5 × 10^5^ particles/mL [140]. Jia et al. introduced an innovative human epidermal growth factor receptor 2 (HER2)-positive EV capture and detection system, termed Magnetic beads@HER2-Exos@HER2-SERS detection nanoprobes. This system enabled efficient extraction and analysis of HER2-positive EVs, and the refined predictive model achieved an accuracy exceeding 0.94 [141]. Wang et al. constructed a surface acoustic wave (SAW)-driven SERS EV capture and analysis chip (SEC-chip). By inducing microfluidic vortices with acoustic waves, the chip efficiently enriched EVs from 5 μL of serum to the detection area within 5 min. It achieved a detection limit as low as five particles per 5 μL, which represents a 100-fold improvement in sensitivity compared with the static incubation method. The characteristic SERS peak intensity showed a good linear relationship across different EV concentrations (SAW-on: R^2^ = 0.995; static control: R^2^ = 0.999). In addition, the chip successfully identified the HER2-positive breast cancer subtype in 12 clinical samples [142]. Zhou et al. constructed a magnetic bead-based capture material based on the coordination between Nd^3+^ and the phosphate groups of a phospholipid membrane. Within 1 h, the material achieved an EV enrichment recovery rate of 46.04%. The relative standard deviations (RSDs) of the signals from three immuno-SERS probes were all below 10%. The quantification of CD63 showed a linear relationship (R^2^ = 0.9749). This method could simultaneously detect three EV-associated markers: CD63, EpCAM, and vascular endothelial growth factor (VEGF). It could also distinguish EVs derived from two breast cancer cell lines, MCF-7 and SK-BR-3 [143].

Subsequent work emphasized paper-based and integrated microfluidic assay formats. To promote related research, Su’s team constructed a novel vertical flow paper SERS biosensor for high-throughput and quantitative analysis of diverse proteins derived from serological EVs [144]. Benefiting from its vertical flow design, the biosensor can avoid false negative signals and achieve multi-index detection of EV-associated proteins on an individual membrane, which significantly boosts analytical efficiency (Figure 6B). One study employed an integrated microfluidic–SERS approach, utilizing tangential flow filtration to isolate EV immunocomplexes for analysis, with an LOD of 2 EVs/mL achieved in blood plasma (Figure 6C) [145]. A separate work adopted a microchip with serpentine microstructures coupled with SERS sensing, accomplishing hands-free analysis of three oncological biomarkers. Its LOD values spanned from 446 to 5460 particles/mL [146].

Other studies expanded specific SERS detection toward multiplexed protein profiling, quantitative immunodetection, and nucleic acid targets. Jin et al. designed a gold-nanowire-based multiplexed extracellular vesicle (GMEX) platform using crossed gold nanowire (AuNW) substrates. The platform employed four SERS tags—naphthalenethiol (NT), 5,5′-dithiobis(2-nitrobenzoic acid) (DTNB), 4-mercaptobenzoic acid (MBA), and 4-bromobenzenethiol (BBT)—to simultaneously detect four surface proteins on colorectal cancer-related EVs: CD24, MUC13, EpCAM, and CD147. It required only 5 μL of sample. The linear correlation coefficients of all targets exceeded 0.95. The detection limit of the system was as low as 10 sEVs per 5 μL. The EV-based colorectal cancer (EVCRC) model, which combined the four markers, achieved a diagnostic accuracy of 92%. This model could identify early-stage colorectal cancer and was used to dynamically monitor postoperative recurrence [147]. Feng et al. used a functionalized membrane modified with amphiphilic–dendritic supramolecular probes (ADSPs) to achieve efficient capture of plasma sEVs. By combining this capture step with three specific SERS nanotags—DTNB, MBA, and thiosalicylic acid (TSA)—they simultaneously quantified three breast cancer-related EV-associated markers: CD9, EpCAM, and HER2. Based on the SERS signals of these markers, they constructed a support vector machine–discriminant analysis (SVM-DA) machine learning model. This model accurately distinguished samples from healthy donors, early-stage breast cancer (Stage 0–II), and metastatic breast cancer (Stage IV), achieving an overall classification accuracy of 89.7% [148]. Zhao et al. developed Ag@Na7PW11O39 nanoclusters that possessed both SERS enhancement and peroxidase-like activity. They modified these nanoclusters with a CD9 antibody for specific quantitative detection of EVs. The linear range for EV detection was 9 × 10^5^ to 1.78 × 10^8^ particles/mL. The LOD was 7.73 × 10^5^ particles/mL. The quantitative correlation coefficient R2 was 0.926. The sensitivity was superior to that of conventional ELISA. The SERS signal in serum from ovarian cancer patients was significantly higher than that from healthy controls (*p* = 0.0018) [149]. In addition, Teng et al. combined rolling circle amplification (RCA) with the clustered regularly interspaced short palindromic repeats (CRISPR)/CRISPR-associated protein 12a (Cas12a) system to construct an ultrasensitive SERS detection platform. This platform used 2,3,5,6-tetrafluoro-4-mercaptobenzoic acid (TFMBA) as a signal tag for the quantitative detection of EV-associated miRNA-21. It showed good linearity over the concentration range of 50 aM to 50 fM (R^2^ = 0.9693). The detection limit was as low as 0.62 aM. The platform could also effectively discriminate single-nucleotide variant sequences. When applied to serum samples from 20 early-stage lung cancer patients and 20 healthy controls, the diagnostic model achieved an area under the curve (AUC) of 1.0 [150].

Microfluidic integration has also been used to combine EV isolation, signal amplification, and SERS detection within a single assay workflow. To improve detection performance, Ma’s group developed a dual-control microchip with enhanced SERS sensing capability. The divided functional chambers supported independent isolation and detection procedures, integrating magnetic manipulation, cascaded DNA signal amplification and electrokinetic enrichment. In practical testing, this microchip was applied to MCF-7-derived EV analysis, offering an LOD of 109 particles/μL and a rapid assay time of 35 min [151]. These integrated formats can simplify multistep specific detection (Figure 6D).

Together, these approaches show how affinity recognition can be combined with different platform formats for specific detection. Label-free SERS provides a complementary route when direct vesicular fingerprints are desired.

### 4.2. Label-Free Detection of EVs Using SERS

In addition to label-based SERS detection based on antigen–antibody recognition, label-free SERS can directly acquire whole-EV fingerprint spectra without complex probe modification and therefore provides another important analytical route.

The distinction between target-specific and non-targeted SERS detection is summarized in Table S1: specificity is determined by selective recognition of a predefined EV-associated marker or subpopulation rather than by the use of a Raman label, and label-free and non-targeted detection should therefore not be regarded as synonymous.

Label-free detection involves directly adsorbing purified EVs onto or bringing them into close proximity to the surface of a SERS substrate to acquire their Raman spectral signals. For instance, Tirinato et al. utilized photolithography to etch regular hexagonal patterns, forming a superhydrophobic surface for detecting EVs isolated from normal colon cells and colon cancer cells. The results revealed that EVs extracted from normal cells exhibited higher peak intensities corresponding to lipid vibrations, whereas those from tumor cell lines showed a richer RNA content [152]. Lee et al. employed sputtering technology to coat a silver film on the surface of 3D nanobowls as the SERS substrate, which was used to detect time-dependent SERS signal changes in EVs derived from ovarian cancer cells [153]. Sivashanmugan et al. assembled Ag nanotubes onto thermally treated ring structures on the surface of Au nanorod arrays to form a plasmonic gap-mode SERS array substrate. They investigated EVs from normal lung cells and cancer cells and found that the stronger SERS signals of lung cancer EVs mainly originated from proteins [139]. Miquel et al. demonstrated that coating a thin Ag layer on conventional recordable discs significantly enhances Raman spectral signals. The study successfully obtained label-free SERS spectra of hemoglobin and EVs, highlighting the biosensing potential of this method [154]. Laura et al. preliminarily investigated compositional differences in EVs derived from ovarian cancer cells under normoxic and hypoxic conditions using SERS technology [155].

A rapid enrichment strategy was also reported for direct SERS quantification. Zheng et al. developed a straightforward approach for the efficient enrichment and SERS quantification of EVs, leveraging the interaction between titanium dioxide (TiO_2_) and the phospholipid bilayer of EV membranes. This method enabled quantitative EV detection from capture to SERS measurement within 10 min, covering five orders of magnitude in the quantification range and achieving an LOD as low as 640 particles/mL. In clinical plasma sample tests, it exhibited strong diagnostic capability in distinguishing cancer patients from healthy individuals, with an AUC of 0.880 [156].

These studies show that label-free SERS can capture broad compositional information, but the resulting multicomponent spectra are more difficult to interpret directly. This helps explain why many recent label-free SERS studies have incorporated AI-assisted analysis, as discussed in Section 4.3.

### 4.3. AI-Assisted Analysis of EV SERS Spectra

Whether label-based or label-free, SERS-based diagnosis ultimately requires effective interpretation of the resulting spectra. Most AI-assisted studies reviewed here use label-free SERS fingerprints because their overlapping, high-dimensional spectral features are difficult to interpret by single-peak analysis. AI functions as a downstream analytical layer, not a third detection mode, and it can be combined with label-based or hybrid assays. Here, label-free refers to the absence of exogenous Raman reporter tags and does not necessarily imply nonspecific EV capture.

**Figure 7:**
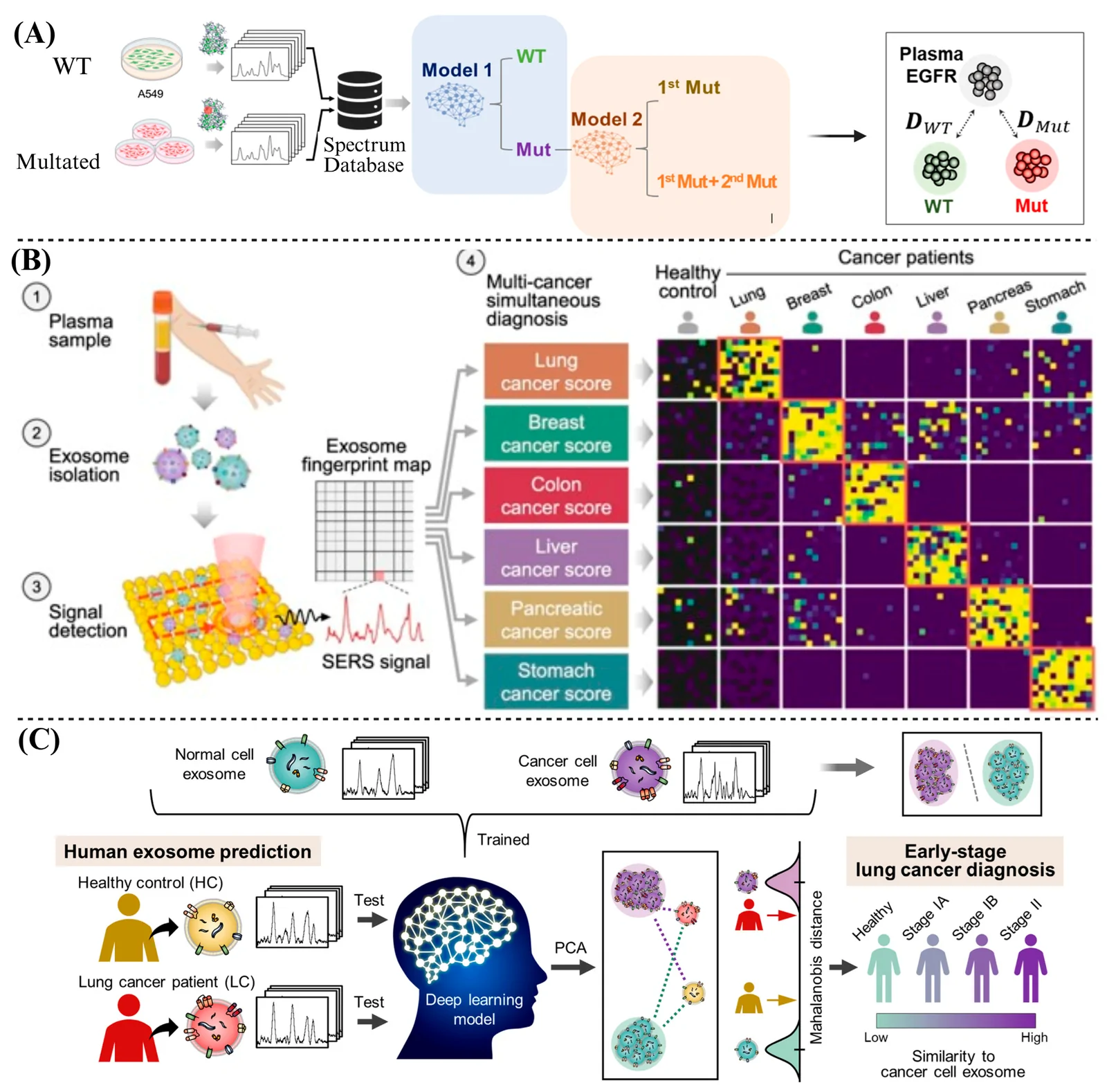
Recent progress in AI-assisted SERS for EV analysis. (**A**) SERS datasets combined with deep learning algorithms for analyzing plasma EV-associated proteins [61]. (**B**) Simultaneous multi-cancer diagnosis utilizing AI-SERS in EV analysis [66]. (**C**) Early-stage lung cancer diagnosis via deep learning-based spectroscopic analysis of circulating EVs [67]. Created in BioRender. Xia, J. (2026). https://BioRender.com/5rmgnbe.

Initial studies mainly employed conventional multivariate methods and deep learning to extract diagnostic features from complex EV-associated SERS spectra. For example, a separate study utilized SERS combined with machine learning techniques for serum EV analysis, which achieved excellent diagnostic sensitivity in identifying breast and cervical cancers [69]. The researchers utilized a 3D ordered array of gold nanoparticles (AuNPs) as the SERS substrate and combined principal component analysis (PCA) with linear discriminant analysis (LDA), enabling the differentiation of EVs derived from normal cells and cancer cells. One investigation realized the integration of Raman spectroscopy with deep learning to detect protein mutations in plasma-derived EVs from patients with lung cancer [61]. This process initially involves acquiring SERS spectra of EV-associated proteins, followed by applying a deep learning algorithm to identify unique protein features (Figure 7A).

Microfluidic integration has enabled EV capture, enrichment, and SERS acquisition to be coupled directly with AI-based classification. In integrated label-free platforms, microfluidics supports EV separation and spectral acquisition, whereas AI performs downstream classification. Chen et al. developed a deep learning-driven microfluidic SERS platform in which anti-CD9-functionalized polystyrene microspheres decorated with gold nanocubes were used for EV capture and enrichment, followed by SERS spectral acquisition and deep learning classification. The platform achieved a maximum trapping efficiency of 85% and distinguished EVs derived from three non-small cell lung cancer (NSCLC) cell lines from those derived from a normal cell line, with an overall accuracy of 97.88% and AUC values above 0.95 for each category [38]. Jin et al. designed a microfluidic chip that integrates tangential flow filtration for size-selective separation and enrichment of plasma EVs. This chip achieved a recovery rate of 82%. Label-free SERS spectra were then acquired using a self-assembled gold nanoparticle array. When combined with a machine learning model, this method achieved noninvasive diagnosis of osteosarcoma with an accuracy of 93%, validating the feasibility of microfluidic label-free SERS in rare tumors [157]. A recent work by Wang et al. reported a novel microfluidic SERS device capable of tracking EV release at the single-cell scale. It is applicable to EV samples from normal breast epithelial cells and multiple breast cancer cell lines, realizing high-precision cell discrimination and early cancer assessment. Moreover, combined with machine learning algorithms, this approach successfully characterized the EV-associated receptor features of distinct breast cancer subtypes post-treatment [158].

At the clinical level, several studies have evaluated AI-assisted SERS using patient-derived EVs for early cancer detection. For example, Shin et al. reported a study that analyzed SERS spectra of EVs using AI. They evaluated the diagnostic accuracy for six types of early-stage cancers. In this study, 520 test samples were used to construct a model for identifying the presence of cancer, with an AUC value of 0.97. Furthermore, the classification of tumor-bearing organs was performed on 278 patients with early-stage cancers, with an average AUC value of 0.945. The final integrated decision model showed a sensitivity of 90.2%, a specificity of 94.4%, and an accuracy of 72% in predicting the tumor type of positive patients [66] (Figure 7B). In addition, Shin et al. utilized deep learning combined with SERS of EVs for the diagnosis of early-stage lung cancer. A deep learning model was trained using SERS of EVs derived from normal and lung cancer cell lines, achieving an accuracy of 95%. Among 43 patients, including those with stage I and II cancers, the deep learning model predicted that 90.7% of the patients had plasma EVs with a higher similarity to lung cancer cell EVs than the average of the healthy control group, and this similarity was proportional to cancer progression. Notably, the model’s AUC for predicting lung cancer patients was 0.912, and for stage I patients, it was 0.910 (Figure 7) [67].

In terms of signal stability and model interpretability, Liu et al. developed a label-free SERS strategy that employs capillary liquid-phase sampling to avoid disruption of the native EV structure during drying. They optimized 44.06 nm AuNPs as the SERS substrate. By combining Bayesian-optimized SVM and convolutional neural network (CNN) dual models and utilizing Shapley additive explanations (SHAP) and partial dependence plot analysis, they revealed the upregulation of collagen and adenine signals in lung cancer EVs, linking these alterations to nucleic acid and metabolic disorders. After validation at three levels—cells, mice, and clinical plasma—the CNN model achieved a diagnostic accuracy of 95.4% for lung cancer [159]. In a different approach to explainable AI, Chen et al. lysed EVs and used a gold nanocube superlattice substrate to simultaneously capture both membrane surface and internal SERS fingerprints. By combining a residual CNN with gradient-weighted class activation mapping (Grad-CAM), they improved the recognition accuracy of lung cancer EVs from 96.35% (using only surface spectra) to 98.95% (after integrating internal signals). Key characteristic peaks revealed by Grad-CAM (e.g., 552, 1302, and 1475 cm^−1^) were associated with mitochondrial abnormalities and altered adenine metabolism, making the model’s decisions interpretable [160].

Beyond early cancer detection, AI-assisted SERS has been extended to identifying tumor origin and characterizing clinically relevant disease phenotypes. One study developed an ultra-small nanoscale 3D SERS sensor for molecular functional analysis of cancer stem cell EVs. The LOD of this SERS sensor was 1 EV/μL. Cancer diagnosis and identification of the primary cancer source can be achieved through cancer stem cell EVs [161]. Wu et al. addressed the challenge of distinguishing radioresistance and recurrence in glioma by combining label-free SERS with proteomics. Using a superhydrophobic chip and a PCA-CNN model, they achieved precise identification of radiation-resistant EVs, with an accuracy of 93.84%. Proteomic analysis revealed that the resistant EVs highly expressed proteins such as aldolase A and glyceraldehyde-3-phosphate dehydrogenase (GAPDH), which are associated with glioma malignancy and poor prognosis. This work extends EV-associated SERS analysis from diagnosis to prognostic prediction of treatment efficacy, providing a molecular basis for personalized radiotherapy [162].

AI-assisted EV-associated Raman analysis has also shown potential for monitoring and predicting therapeutic response. A study by Jia et al. employed Raman spectroscopic analysis to characterize molecular changes in EVs isolated from the serum of HER2-positive breast cancer patients, before and following two rounds of neoadjuvant treatment. By leveraging machine learning algorithms, a predictive model was developed, with its AUC reaching above 0.89 [141].

More recent studies have further expanded AI-assisted SERS toward patient-level diagnosis, interactive spectral interpretation, and pan-cancer screening. Wang et al. performed diagnosis of pancreatic cancer (PaC) patients via AI-based spectral analysis of EVs from 149 serum samples, achieving an AUC of 0.96 and successfully classifying 24 cases of early-stage PaC. Furthermore, the maximum diagnostic positivity rate among 161 non-pancreatic cancer cases was 4.44%, confirming the specificity of the model [163]. Moreover, Yang et al. developed an AI agent centered on large language models, named ChatEV, that created an interactive and user-friendly system for clinical spectroscopic analysis and diagnosis. This system, which includes deep learning-based analysis of the Raman fingerprints of EVs derived from hepatocellular carcinoma, achieved accuracies of 95.8% for cell-derived EVs and 94.1% for 165 clinical samples [164]. For pan-cancer screening, Zhang et al. fabricated a CP05 peptide-modified gold nanoisland (AuNIS) SERS chip to specifically capture plasma CD63-positive EVs. They built a hybrid CNN–multilayer perceptron (MLP) AI model and included plasma samples from 200 healthy individuals and 455 patients with ten cancer types (including lung, breast, stomach, pancreas, and ovary). The accuracy for healthy/tumor binary classification was 97.4%, and the detection accuracy for early-stage tumors (T1/T2) reached 97.08%. For the ten-cancer multiclass classification, the accuracy was 93.89%. Combined metabolomic and spectral analysis revealed that the peak at 1080 cm^−1^, assigned to deoxyadenosine triphosphate (dATP), was stably upregulated in all tumor EVs, acting as a broad-spectrum pan-cancer molecular fingerprint. This finding provides a unified biomarker target for simultaneous multi-cancer early screening [165]. He et al. proposed a fusion strategy at the experimental design level to address the interpretability of label-free SERS. In this study, while acquiring label-free SERS fingerprints of plasma EVs from ovarian cancer patients, they introduced antibodies labeled with polydiacetylene Raman tags targeting cancer antigen 125 (CA-125), human epididymis protein 4 (HE4), and carbohydrate antigen 19-9 (CA-19-9). The characteristic signals from the tags were then integrated with the label-free whole-component spectra and fed into a machine learning model. After combining the two signal modalities, the SVM achieved a classification accuracy exceeding 95%, outperforming either modality alone. By chemically linking spectral features to known biomarkers through Raman tags, this strategy fundamentally endows label-free SERS fingerprints with explicit molecular interpretation and provides experimental assurance for the clinical credibility of SERS diagnostics [166].

AI-assisted analysis facilitates the extraction of diagnostic information from complex EV-associated SERS spectra and has supported applications in cancer detection, molecular subtyping, and treatment response assessment. Within this section, the majority of studies rely on label-free spectral fingerprints; however, antibody-assisted capture and hybrid strategies have also been explored to improve analytical specificity and molecular interpretability. Several methodological limitations should be noted. Some reported high accuracies were obtained with small cohorts or spectrum-level splitting, which risks overfitting. Most studies employed healthy donor controls, which likely overestimates specificity relative to clinically relevant benign or inflammatory disease controls. Nearly all validations were internal, and external multi-institutional testing remains rare. Clinical translation will require not only standardized sample preparation, reproducible substrates, and reproducible acquisition protocols but also rigorous patient-level validation, external validation, and transparent reporting of AI model development and performance.

Table S2 compares the studies discussed in Section 4 and shows that label-based, multiplexed, and microfluidic platforms emphasize target specificity and workflow integration, whereas label-free and AI-assisted approaches offer broader molecular fingerprinting but depend more strongly on standardized sample preparation, reproducible substrates, and robust spectral analysis. Table S3 further shows that high sensitivity, specificity, accuracy, or AUC values were frequently reported from small cohorts with limited patient-level or external validation and should therefore be interpreted separately from analytical LOD.

Previous reviews have established the analytical basis of EV-based SERS, with different emphases on sensing formats, substrate engineering, and spectral analysis. As summarized in Table 2, the present review makes a distinctive contribution by providing an integrated translational assessment. This assessment links EV isolation, storage, and other preanalytical factors to the reproducibility of SERS fingerprints and critically evaluates clinical evidence and AI validation, including patient-level data splitting, batch effects, spectral metadata, and external validation. The review extends the discussion beyond cancer detection to treatment monitoring, recurrence assessment, and prognosis.

## 5. Conclusions and Perspectives

**Figure 8:**
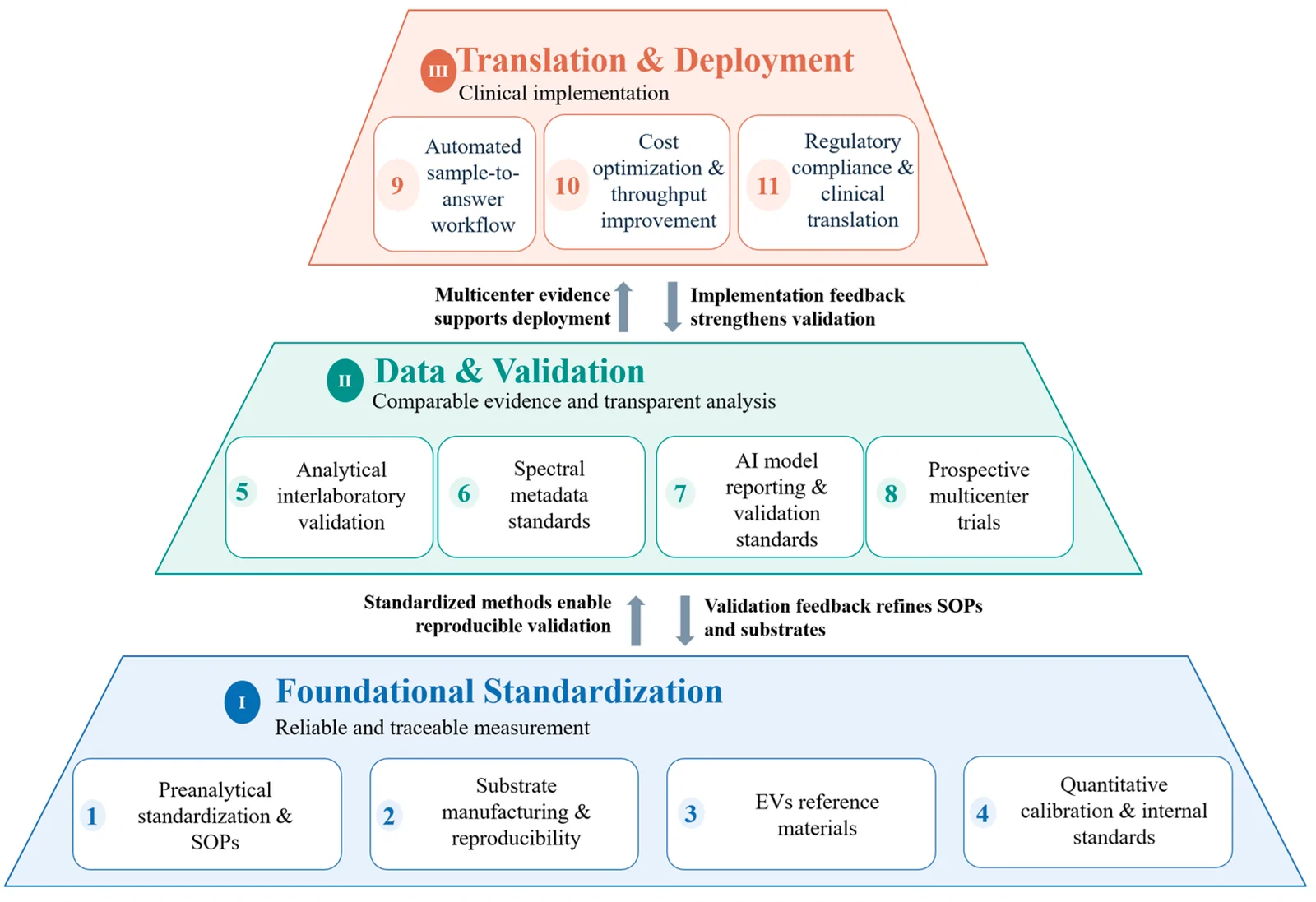
A roadmap for the clinical translation of EV-based SERS diagnostics.

The limitations of tissue biopsy have become increasingly apparent in precision oncology because of its invasiveness, restricted sampling frequency, and limited ability to capture tumor heterogeneity. EVs circulate stably in accessible body fluids and carry molecular information associated with tumor development and progression, supporting their potential as diagnostic and prognostic biomarkers [170,171]. This review systematically summarizes the application of EV-based SERS in cancer diagnosis. Specifically, it covers EV isolation, characterization, and storage. It also discusses the fundamental principles of SERS and the Raman spectral characteristics of EVs. Furthermore, it reviews recent advances in specific label-based detection, label-free Raman fingerprinting, and AI-assisted spectral analysis. Collectively, the reviewed studies demonstrate the potential of EV-based SERS for early cancer detection, molecular subtyping, treatment response assessment, and prognostic monitoring. To summarize the key priorities for clinical translation, Figure 8 presents a three-phase roadmap for EV-based SERS diagnostics. The bidirectional arrows indicate continuous feedback and optimization across the three phases.

Despite this progress, the heterogeneity in EV size, composition, and surface proteins remains a major challenge for reproducible SERS analysis. Variations in sample collection, processing, isolation, storage, and freeze–thaw exposure may influence EV purity and composition and consequently alter the measured SERS spectra. Future studies should therefore standardize these preanalytical procedures and report them in sufficient detail. Furthermore, biological and demographic variables—such as age, sex, smoking status, and comorbidities—may also influence EV-associated cargo and consequently alter SERS spectra, yet they have seldom been controlled or reported. Future diagnostic models should therefore collect and adjust for these potential confounders. SERS substrates must also provide not only strong signal enhancement but also adequate spatial uniformity, batch-to-batch reproducibility, storage stability, and controlled sample deposition to minimize effects such as nonuniform hotspot distribution and coffee-ring formation. The development of suitable reference materials, internal standards, and quantitative calibration procedures will be important for comparing results across substrates and instruments. Analytical limits of detection should also be distinguished from clinical diagnostic performance because high sensitivity in purified or spiked samples does not necessarily translate into reliable patient classification. In addition, most reported diagnostic models have been evaluated using healthy donor controls, which may overestimate specificity compared with clinically representative controls, such as patients with benign or inflammatory diseases. Future studies should incorporate more challenging control groups to better assess real-world diagnostic utility.

The growing volume and complexity of EV-associated SERS data have driven the use of machine learning and deep learning. Although these approaches can extract diagnostic information from overlapping spectral features, their reported performance may be affected by small cohort sizes, spectrum-level (rather than patient-level) data splitting, data leakage, and substrate or instrument batch effects. Future studies should provide complete spectral metadata and clearly report preprocessing procedures, model architecture, validation strategies, and the number of independent patients per dataset. Interlaboratory comparisons and prospective multicenter studies will be needed to determine whether the spectral features and AI models remain reproducible across different patient populations, operators, substrate batches, and Raman instruments.

For clinical translation, EV isolation, substrate loading, spectral acquisition, quality control, and AI analysis should ultimately be integrated into automated sample-to-answer workflows. In addition to analytical and clinical performance, factors such as manufacturing reproducibility, throughput, operational complexity, cost, and regulatory requirements must be considered during platform development. In the longer term, combining SERS with imaging and omics technologies may improve the molecular interpretation of Raman fingerprints, facilitate the discovery of potential cancer biomarkers, and provide further insights into disease progression and therapeutic response. Coordinated progress in standardization, validation, automation, and clinical evaluation will be essential for translating EV-based SERS from laboratory research into reliable cancer diagnostic applications.

## Figures and Tables

**Table 2:** Comparison of the scope and emphasis of representative reviews on SERS-based extracellular vesicle analysis.

Comparison Item	Guerrini et al. (2021) [167]	Li et al. (2022) [168]	Zheng et al. (2022) [169]	Liu et al. (2024) [39]	Present Review
Overall focus	SERS principles; direct and indirect EV analysis	Substrate design and functionalization; immunoassays and integrated detection	Label-free and label-based assays; signal analysis and engineered EV monitoring	Label-free SERS for cancer diagnosis	Preanalytical handling, spectral reproducibility, clinical evidence, and AI validation
Detection strategies	Direct label-free fingerprinting and indirect SERS-tag assays	Label-free and SERS-tag assays, with emphasis on immunoassays	Direct label-free and label-based SERS; substrates and nanoprobes	Label-free SERS; SERS-tag assays discussed for comparison	Label-based target detection and label-free fingerprinting, microfluidic and AI-assisted formats
EV isolation and characterization	Dedicated overview; isolation purity identified as a source of spectral variability	Separation methods reported mainly within individual assay designs	Purification and characterization subsection; centrifugation and trypsin treatment considered	Comparison of common methods; emphasis on EV size, concentration, and sample source	Isolation-dependent purity, recovery, and matrix carryover considered in relation to SERS measurements
EV storage and preanalytical factors	Sample preparation, adsorption, and drying discussed; no dedicated EV storage analysis	No dedicated EV storage analysis; storage discussion concerns nanoparticle and reporter stability	No dedicated discussion of EV storage or freeze–thaw effects	No dedicated discussion of EV storage or freeze–thaw effects	Storage temperature, medium, freeze–thaw exposure, and residual matrices linked to SERS variability
Substrate-related issues	Hotspot geometry, adsorption, and signal variability; need for robust, scalable substrates	Substrate materials, functionalization, stability, and multistage signal enhancement	Substrate fabrication, signal enhancement, and nanoprobe assembly	Enhancement factor, uniformity, reproducibility, stability, and biocompatibility	Different substrates (e.g., Au@Ag, nanobowtie, and gap mode structures) govern the selectivity of SERS enhancement; the sample deposition method affects hotspot uniformity and signal reproducibility
Clinical translation	Patient studies plus discussion of scale-up, cost, portability, automation, and isolation standardization	Clinical examples and microfluidic integration; limited appraisal of study design	Clinical examples; EV purity standards, portable instrumentation, automation, and assay integration	Diagnostic studies summarized; larger cohorts, standardized workflows, and lower-cost instrumentation recommended	Analytical LOD distinguished from clinical performance; cohort and validation design, batch effects, automation, cost, and regulatory translation discussed
Spectral and AI analysis	PCA, PLS-DA, and an early deep learning study; validation methodology is not a main focus	PCA and multivariate examples; no separate AI analysis section	PCA, LDA, PLS-DA, deep learning, and classifier comparisons	Peak analysis, PCA, conventional classifiers, and deep learning reviewed in dedicated sections	Separate AI section covering patient-level splitting, leakage, metadata, batch effects, interpretability, external validation, and reporting
Treatment monitoring and prognosis	Melanoma EV phenotyping during BRAF inhibitor treatment; wider clinical potential noted	Monitoring and prognostic potential noted; diagnosis is the principal application	BRAF treatment phenotyping and engineered EV tracking; progression and recurrence discussed as potential applications	Selected drug response and radioresistance studies; diagnosis remains the main focus	Treatment response, radioresistance/recurrence, and prognostic applications covered; clinical evidence remains preliminary

Note: Entries compare relative emphasis rather than absolute presence or absence; “not a main focus” does not exclude brief mention. EV, extracellular vesicle; LDA, linear discriminant analysis; LOD, limit of detection; PCA, principal component analysis; PLS-DA, partial least-squares discriminant analysis; SERS, surface-enhanced Raman scattering.

## Data Availability

No new data were created or analyzed in this study. Data sharing is not applicable to this article.

## Supplementary Materials

The following supporting information can be downloaded at https://www.mdpi.com/article/10.3390/bios16090464/s1: Table S1: Comparison of target-specific and non-targeted extracellular vesicle detection using SERS; Table S2: Comparative analytical characteristics of EV-based SERS studies for cancer detection across label-free, label-based, microfluidic, multiplexed, and AI-assisted strategies; Table S3: Clinical cohorts, validation strategies, diagnostic performance, and major limitations of EV-based SERS studies for cancer diagnosis.
