# Supplementary material for: Advances in Extracellular Vesicle-Based Surface-Enhanced Raman Spectroscopy for Cancer Diagnosis
Source: Biosensors (Basel). 2026 Aug 26;16(9):464. doi: 10.3390/bios16090464 (PMC13604868; doi:10.3390/bios16090464)
Supplement: Supplementary file 1 [file biosensors-16-00464-s001.zip › biosensors-4447980-supplementary.pdf]

Supplementary Materials

Advances in Extracellular Vesicle-Based Surface-Enhanced Raman Spectroscopy for Cancer Diagnosis

Shuyuan Zhao [1], Wen Lei [2], Juan Li [2] and Jingjing Xia [2,*]

[1] Department of Thoracic and Abdominal Radiation Oncology, Cancer Hospital of Xinjiang Medical University, No. 789 Suzhou Road, Urumqi 830011, China
[2] College of Life Science and Technology & School of Pharmaceutical Sciences and Institute of Materia Medica, Xinjiang University, No. 777, Hua Rui Street, Shui Mo Gou District, Urumqi 830017, China
* Correspondence: xiajing94@xju.edu.cn

Table S1. Comparison of target-specific and non-targeted extracellular vesicle detection using SERS.

Comparison aspect	Target-specific EV detection	Non-targeted (nonspecific) EV detection
Defining basis	Selects predefined EV-associated biomarkers or subpopulations through target recognition elements before or during SERS readout.	Measures the overall isolated or captured EV population without disease- or subtype-selective recognition.
Typical workflow	Selective capture and detection, often using a sandwich format: capture support–EV–SERS nanotag.	Broad EV isolation or capture, followed by direct interaction with a plasmonic substrate and spectral acquisition.
Recognition mechanism	Antibody–antigen, aptamer–target, peptide–receptor, nucleic acid hybridization, or CRISPR-based recognition.	No disease- or subtype-selective recognition step; retention is commonly based on adsorption, electrostatic interactions, or surface wettability.
SERS readout	Usually generated by Raman reporter nanotags; label-free SERS may also be used after selective capture.	Usually based on intrinsic fingerprints of EV-associated lipids, proteins, and nucleic acids; a label alone does not confer target specificity.
Typical output	Marker-specific abundance, target-positive EV counts, or multiplexed phenotypes, often with quantitative calibration.	Composite spectral patterns for sample classification or biomarker discovery; individual bands may lack unique molecular assignments.
Main advantages	High analytical selectivity and sensitivity; supports quantitative and multiplexed detection of predefined targets.	Requires no disease-specific recognition probe or prior biomarker selection; retains broad molecular information and simplifies assay design.
Main limitations	Restricted to predefined targets and dependent on reagent affinity, epitope availability, cross-reactivity, and capture efficiency.	Sensitive to co-isolates and sample, substrate, instrument, and batch variability; data-driven classification requires rigorous patient-level and external validation.

Note: Specificity is defined by selective recognition of a predefined EV-associated marker or subpopulation, not by the presence of a Raman label. Label-free and non-targeted detection are therefore not synonymous; either readout can be combined with selective or non-selective capture, depending on assay design. AI-assisted classification does not itself confer molecular specificity.

Table S2. Comparative analytical characteristics of EV-based SERS studies for cancer detection across label-free, label-based, microfluidic, multiplexed, and AI-assisted strategies.

Study category	Cancer type	Sample source	EV isolation method	SERS substrate	Recognition chemistry	Sample volume	Assay time and LOD	Detection strategy	Reference

Label-free	Colon cancer	CCD841-CoN and HCT116 cell culture supernatants	ExoQuick, differential centrifugation, and 0.45-µm filtration	AgNP-coated superhydrophobic silicon micropillars	Label-free intrinsic lipid and RNA fingerprints	10 µL (0.2 ng/mL)	- and tested at 0.2 ng/Ml	Superhydrophobic concentration and label-free SERS; discrimination of cancer and normal cell EVs	[152]
	Ovarian cancer	SKOV3 cell culture supernatant	TEIR kit or differential ultracentrifugation	Silver-coated 3D PDMS nanobowls	Label-free intrinsic EV fingerprint	20 µL	Monitoring at 0, 1, and 3 h; 10 s/spectrum and lowest tested concentration: 580 fM	Single-EV capture, time-resolved SERS, and PCA	[153]
	Lung cancer	Normal lung cells and PC-9, H1975, and HCC827 cell culture supernatants	Ultracentrifugation	Au nanorod/Ag nanocube bimetallic gap substrate	Label-free intrinsic biochemical fingerprint	5 µL (1.1×10^6–1.2×10^8 particles/mL)	10 s/spectrum; 15–45 min incubation and approximately 10^4–10^5 particles/mL	High-enhancement label-free SERS; discrimination of normal and lung cancer EVs	[139]
	Lung cancer	A549 cell culture supernatant	Ultracentrifugation	Ag-sputtered CD-R/DVD-R grooved substrate	Label-free intrinsic EV/protein fingerprint	5 µL	10 s/spectrum and -	Low-cost optical disc SERS platform for point-of-care testing	[154]
	Ovarian cancer	Normoxic and hypoxic A2780 cell culture supernatants	Differential ultracentrifugation and 0.22 µm filtration	CaF_2 substrate with 50-nm GNP enhancement	Label-free intrinsic EV fingerprint	10 µL EVs; an additional 10 µL GNPs for SERS	Raman: 20 s/spectrum; SERS: 10 s/spectrum and -	Raman/SERS with PCA-DFA to distinguish normoxic and hypoxic EVs	[155]
Label-based (multiplexed)	Ovarian and breast cancers	Sera from patients and healthy controls	Ultracentrifugation	$Ag@Na_7PW_{11}O_{39}$ nanozyme SERS tags	Antibody recognition: anti-CD9 and anti-TSG101	-	Catalysis: 30 min and CD9: 7.73×10^5; TSG101: 4.05×10^5 particles/mL	SERS-NELISA with nanozyme-catalyzed signal amplification	[149]
Label-based	Early-stage lung cancer	Serum from 20 patients and 20 healthy controls	SEC; kit-based extraction of EV-associated miRNA	AuNP SERS tag/ssDNA/magnetic bead complex	DNA hybridization with RCA and CRISPR-Cas12a	500 µL serum; 100 µL complex	Approximately 3–4 h and 0.62 aM (miRNA-21)	RCA–Cas12a cascade amplification and SERS quantification after magnetic separation	[150]
	Breast and pancreatic cancers	Clinical plasma; model EVs in urine	HPL-SEC; Mini-SEC with $Fe_3O_4@TiO_2$ enrichment	$Ag@NTP@TiO_2$ SERS tags	TiO_2–phosphate coordination	50 µL model sample; 10 µL diluted plasma	10 min; clinical workflow: 25 min and 640 particles/mL	$Fe_3O_4@TiO_2$ capture and $Ag@NTP@TiO_2$ tag-based sandwich assay	[156]
Microfluidic (label-based)	HER2-positive breast cancer	SKBR3 and HeLa cell culture supernatants; patient serum	On-chip SAW enrichment via a CD63 aptamer	AgNP probes on an Au-coated polyimide chip	CD63 capture aptamer and HER2 detection aptamer	5 µL	SAW enrichment: 5 min; total: approximately 65 min and 1 particle/µL	SAW microfluidic enrichment and in situ SERS quantification	[142]
	Breast cancer	MCF-7 and MCF-10A supernatants; spiked serum	On-chip capture with MUC1-aptamer magnetic beads	AuNP–DNA SERS probes (RS-1/RS-2)	MUC1 aptamer with DNAzyme/hairpin strand displacement	Microliter scale	35 min and 10.9 particles/µL	Magnetic separation/electrokinetic enrichment with bidirectional SERS signal amplification	[151]

	Osteosarcoma	143B, HOS, and MG63 supernatants; plasma from 20 patients and 20 controls	Microfluidic tangential flow filtration; PCTE membrane capture	AuNP SERS tags on a PCTE membrane	Anti-CD63, anti-CD9, anti-VIM, and anti-EpCAM	50 µL plasma; 500 µL supernatant	Approximately 5 h and 2 particles/µL	Microfluidic isolation, membrane-based SERS, and PLS-DA	[145]
Microfluidic (label-based, multiplexed)	Lung cancer	NCI-H226, A549, and BEAS-2B cell culture supernatants	CD63 magnetic bead capture in a serpentine microfluidic chip	50 nm AuNP tags and 150 nm magnetic beads	Anti-CD63 capture; CD63, PD-L1, and EGFR probes	50 µL supernatant; 25 µL each of beads and tags	Approximately 1 h and CD63: 5.46×10^3; PD-L1: 2.44×10^3; EGFR: 4.46×10^2 particles/mL	One-step microfluidic sandwich SERS with multiphenotypic detection	[146]
	Breast cancer	Single-cell cultures of MCF-7, MDA-MB-231, and MCF-10A	In-droplet capture with immunomagnetic beads	56 nm AgNP-encoded SERS probes	Sandwich immunorecognition of CD44, PD-L1, and CEA	2.1 nL/droplet; 50 µL for bulk validation	Incubation: 3 h; 5 s/spectrum and -	Single-cell droplet microfluidics, three-marker SERS, and RF/t-SNE	[158]
	Ovarian cancer	OVCAR3 and A2780 supernatants; sEV-spiked plasma	Differential ultracentrifugation	AuNP SERS tags and antibody-conjugated magnetic beads	Anti-CD9/CD63/CD81 capture; anti-EpCAM/CA125/CD24 detection	100 µL sEVs; 50 µL tags	Approximately 2 h and 1.5×10^5 particles/µL	Magnetic bead sandwich SERS for multiplex EpCAM, CA125, and CD24 detection	[140]
	Breast cancer	MCF-7, SK-BR-3, and MCF-10A cell culture supernatants	Nd^{3+}-mediated coordination capture on magnetic beads	AgNP-encoded SERS probes	Nd^{3+}–phosphate coordination; anti-CD63, anti-EpCAM, and anti-VEGF	0.1–4 mL supernatant	Approximately 3–4 h and response range: 10^5–10^7 particles	Rare earth magnetic enrichment and multiplex SERS of CD63, EpCAM, and VEGF	[143]
Multiplexed (label-based)	Breast cancer (luminal A/B, HER2-positive, and TNBC)	Patient serum	SEC for cell samples; ExoQuick for serum	Au nanostar@SiO_2/p-NTP tags	Anti-MUC1, anti-HER2, and anti-CEA with a CD63 aptamer	75 µL serum	15–20 min and 1.0–2.9×10^7 particles/mL	Paper-based vertical flow sandwich SERS for three-marker breast cancer subtyping	[144]
	Colorectal cancer	CRC and normal-cell supernatants; training and validation plasma cohorts	Dual-mode SEC; immunocapture on Au nanowires	Crossed Au nanowire substrate and Au@SiO_2 tags	CD9/CD63/CD81 capture; CD9/CD63/CD81/EpCAM detection probes	5 µL sEVs; 500 µL plasma	SEC: approximately 2 h; SERS: 10 min and EpCAM: 0.8; CD63: 1.7; CD81: 1.5; CD9: 2.0 particles/µL	GMEX multiplex SERS with a composite biomarker score for recurrence diagnosis	[147]
	Breast cancer (DCIS, early-stage, and metastatic)	SKBR3 and MCF7 supernatants; plasma from 58 patients	In situ capture on an ADSP-modified nitrocellulose membrane	Approximately 56 nm AuNP SERS tags	Anti-CD9, anti-EpCAM, and anti-HER2	10 µL plasma; 2 µL for detection	-	High-throughput membrane array multiplex SERS for stage classification	[148]
Multiplexed (label-free, label-based, AI-assisted)	Ovarian cancer	Serum	SEC (qEV35)	Gold nanoisland glass substrate with polyyne Raman tags	Anti-CA125, anti-HE4, and anti-CA19-9; intrinsic EV fingerprints	150 µL serum	Approximately 1 h and -	Label-free fingerprinting plus multiplex SERS with three polyyne tags and SVM	[166]
AI-assisted (label-free, label-based)	HER2-positive breast cancer	Patient serum before treatment and after two treatment cycles	Ultracentrifugation; EasySep HER2 immunomagnetic sorting	Au nanostar@SiO_2/4-NTP	Label-free fingerprint; anti-HER2 recognition	Label-free Raman: 10 µL; SERS: not reported	1 s/spectrum; total workflow: several hours and -	Raman and SERS with PCA–LDA–SVM for treatment-response prediction	[141]

AI-assisted (label-based)	NSCLC with EGFR mutations	Five cell line supernatants; plasma from healthy, EGFR wild-type, and mutant cohorts	SEC; lysis to release EV-associated proteins	Anti-EGFR glass substrate with 100 nm GNPs	Anti-EGFR antibody capture	500 µL plasma	1 s/spectrum; 400 spectra/sample and -	Targeted SERS with a CNN to distinguish wild-type/mutant and primary/resistance mutations	[61]
	NSCLC	BEAS-2B and three NSCLC cell line supernatants	ExoQuick-TC	PS-AuNC capture probe (PACD)	Anti-CD9	0.5 mL	Approximately 1 h; 3 s/spectrum and tested at 2.64×10^{5}–1.64×10^{6} particles/mL	Microfluidic label-free SERS with ResNet/Grad-CAM for subtype classification	[38]
AI-assisted (label-free, microfluidic)	Osteosarcoma	Three osteosarcoma and HeLa supernatants; patient and control plasma	Microfluidic TFF with an 80 nm PCTE membrane	78 nm GNP monolayer array	Label-free intrinsic EV fingerprint	500 µL raw sample; 1 µL EVs for detection	10 s × 10 spectra/sample and -	Microfluidic purification and label-free SERS with random forest classification	[157]
	Ten cancer types (pan-cancer)	Serum	On-chip capture	AuNIS@CP05 with SH-PEG	Specific CP05–CD63 binding	200 µL diluted serum	Capture: 7 h and R6G enhancement factor: approximately 9×10^{4}	Integrated SERS chip with CNN–MLP for pan-cancer classification	[165]
	Six early-stage cancers	Blood-derived EVs from cell line and clinical samples	-	-	Label-free intrinsic Raman features	-	-	Label-free SERS with CNN/SVM/MLP for cancer screening and primary-site identification	[66]
	Lung adenocarcinoma (stages IA–IIB)	Five cell line supernatants; patient and control plasma	SEC (2B/6B Sepharose and Sephacryl S-200)	Aggregated 100 nm GNP substrate	Label-free protein, nucleic acid, and lipid fingerprints	3 mL blood; 50 µL EVs for detection	10 s/spot; 100 spots/sample and -	Label-free SERS with ResNet and cell-to-clinic transfer learning	[67]
AI-assisted (label-free)	Early-stage NSCLC (stages I–II)	Cell culture supernatants, mouse plasma, and clinical plasma	Ultracentrifugation, magnetic bead kit, or TFF	44.06 nm AuNP colloid	Label-free intrinsic EV fingerprint	3 µL EVs and 3 µL AuNPs	30 s/spectrum and R6G enhancement factor: 1.04×10^{4}	Liquid-phase label-free SERS with optimized SVM/CNN and multilevel validation	[159]
	Lung cancer (NSCLC)	A549, PC-9, NCI-H226, and BEAS-2B cell culture supernatants	ExoQuick-TC	Au nanocube superlattice	Label-free intrinsic EV fingerprint	5 mL supernatant; 2 µL EVs for detection	Precipitation: ≥12 h; 8 s/spectrum and R6G: 10^{-8} M	SERS imaging with ResNet/Grad-CAM for surface and internal biomarker mapping	[160]
	Breast, lung, and colorectal cancers	Tumor cells, cancer stem cell spheres, and a small plasma cohort	Differential ultracentrifugation	3D interconnected TiO_2 nanonetwork	Label-free intrinsic EV fingerprint	5 µL EVs	10 s/spectrum; three replicates and 10 EVs/10 µL	TiO_2-based label-free SERS with ANN for diagnosis and tissue-of-origin classification	[161]

Glioblastoma (radioresistant/radiosensitive)	U251 RR and RS cell culture supernatants	-	Dodecanethiol-functionalized hydrophobic self-calibrating 2D substrate	Label-free intrinsic EV fingerprint	-	-	Label-free SERS with PCA-CNN; PCA-LDA used as a comparator	[162]
Pancreatic cancer	Serum; other cancer types used for specificity testing	exoEasy Maxi	Hierarchical MLA/AgNW/AgNP substrate	Label-free intrinsic EV fingerprint	500 μL serum	10 s/spectrum and R6G: 10^{-9} M	Label-free SERS with CNN-based diagnosis	[163]
Hepatocellular carcinoma	Clinical plasma; normal hepatocytes and seven liver cancer cell line supernatants	Ultracentrifugation	Self-assembled AuNP array	Label-free intrinsic EV fingerprint	-	- and R6G CV: 4.70%	Label-free SERS with an FFT–Transformer and a large language model agent	[164]
Breast and cervical cancers	H8, HeLa, and MCF-7 supernatants; serum from 17 subjects	Commercial precipitation kit	Three-layer 3D AuNP nanofilm	Label-free intrinsic EV fingerprint	20 μL (approximately 10^{8} particles/mL)	15 s/spectrum and -	3D SERS with PCA-LDA for cancer-type classification and dynamic treatment monitoring	[69]

Table S3. Clinical cohorts, validation strategies, diagnostic performance, and major limitations of EV-based SERS studies for cancer diagnosis.

Cancer Type	Cohort Size	Validation Strategy	Clinical Performance	Major Limitations	Reference
HER2-positive breast cancer	56 patients: label-free Raman, n=28; HER2-MEDN SERS, n=28.	Each arm split 70:30 for training/testing; SVM and ROC analysis.	Label-free: accuracy >90%, AUC >0.89; SERS: accuracy >94%, AUC >0.90.	Limited sample size and lack of an independent external cohort.	[141]
Osteosarcoma	40 plasma samples: 20 patients and 20 healthy controls.	Training, n=30; test, n=10; PLS-DA with 10-fold CV in training.	Training AUC=0.971; test sensitivity 100%, specificity 90%, accuracy 95%.	The validation cohort is relatively small.	[145]
Colorectal cancer	113 plasma samples: training, n=53; independent validation, n=60; 9 postsurgical follow-ups.	Logistic-regression EV_CRC score; independent validation and longitudinal monitoring.	Training: AUC=0.97, sensitivity 97%, specificity 81%, accuracy 92%; validation: AUC=0.96, sensitivity 90%, specificity 80%, accuracy 87%.	The cohort size is limited.	[147]
Breast cancer (stages 0–IV)	Plasma n=58: healthy 8, stage 0 8, I 16, II 16, IV 10; independent prediction set n=8: healthy 2, early-stage 4, late-stage 2.	K-means, SVM-DA, and random forest; independent prediction-set assessment.	Main cohort accuracy 89.7%; independent prediction set accuracy 87.5%.	Uneven subgroup distribution, and the prediction set is relatively small.	[148]

Early-stage lung cancer	40 serum samples: 20 patients and 20 healthy controls.	Whole-cohort ROC analysis; qRT-PCR comparison and DeLong test; no independent set.	SERS: AUC=1.00, sensitivity 100%, specificity 100%; qRT-PCR: AUC=0.9875.	Evaluated on the same cohort; no independent validation set.	[150]
Breast and cervical cancers	17 serum samples: breast cancer 7, cervical cancer 5, healthy controls 5.	LDA trained on cell line EVs and applied to clinical samples.	Accuracy: breast cancer 100%, cervical cancer 100%, healthy controls 80%; overall 93.3%.	The cohort size is limited.	[69]
EGFR-mutant NSCLC	61 plasma samples: healthy 19, EGFR wild-type 16, EGFR-mutant 26; 3 paired follow-ups.	Cell line CNN features applied to clinical similarity classification; no independent clinical cohort.	Clinical accuracy 93%; reported sensitivity and specificity 100%; secondary mutation score increased ~2.62-fold in 3 paired cases.	No independent clinical validation cohort.	[61]
Osteosarcoma	43 plasma samples: 28 patients and 15 healthy controls.	Training: 22 patients+12 controls; validation: 6+3; RF, SVM, and PLS-DA compared.	RF validation: accuracy 93%, sensitivity 94%, specificity 89%, AUC=0.92.	The sample size is limited.	[157]
Lung adenocarcinoma (stages IA–IIB)	63 plasma samples: 43 patients and 20 healthy controls.	Cell model features; Mahalanobis distance classification of clinical samples; algorithm comparison.	Overall AUC=0.912, sensitivity 84%, specificity 85%; stage I AUC=0.910; stage IA AUC=0.840.	The clinical sample size is limited.	[67]
Early-stage NSCLC (stages I–II)	18 clinical samples: 10 patients and 8 healthy controls.	Hierarchical cell–animal–clinical validation; 6:1:3 split; 5-fold CV; SHAP/PDP.	Clinical samples: SVM accuracy 91.5%, AUC=0.9714; CNN accuracy 95.4%, AUC=0.9916.	The clinical sample size is small.	[159]
Breast, lung, and colorectal cancers	Small pilot clinical cohort; size and composition not reported.	GAN augmentation; 80:20 split; independent blinded test; PLS-DA comparison.	Cancer/healthy sensitivity and specificity 100%; pairwise tissue-of-origin accuracy 100%; multiclass accuracy 79%.	The cohort size and composition are not clearly reported.	[161]
Breast and pancreatic cancers	22 plasma samples: 13 patients (breast 7, pancreatic 6) and 9 healthy controls.	Matrix effect and repeatability assessment; whole-cohort ROC; no independent set.	Cancer versus healthy controls: AUC=0.880; group difference $p<0.01$.	The sample size is small.	[156]
Pancreatic cancer	Main cohort n=149: 72 patients and 77 healthy controls; 161 other cancers for specificity testing.	4:1 training/test split; augmentation; comparison with RF and CA19-9; cross-cancer specificity testing.	Test AUROC=0.96; early-stage sensitivity 100%, specificity 98.7%; maximum false positive rate in other cancers 4.44%.	Further validation in prospective multicenter cohorts is still needed to confirm its clinical translation potential.	[163]
Hepatocellular carcinoma (including AFP-negative cases)	165 plasma samples: 125 patients and 40 healthy controls.	Clinical validation; CNN versus ViT; AFP-negative subgroup and metabolomic validation.	HCC/healthy accuracy 94.1%; AFP-negative HCC/healthy accuracy 87.5%.	Lack of independent external validation.	[164]

Pan-cancer (10 cancer types)	655 serum samples: 455 patients and 200 non-cancer controls.	5-fold CV; cancer screening, early detection, and 10-cancer classification; metabolomic and interpretability analyses.	Cancer/non-cancer: accuracy 97.4%, AUC≈0.99; early cancer: accuracy 97.08%, AUC=0.99; 10-cancer: accuracy 93.89%, sensitivity 93.1%, specificity 99.03%.	As a single-center exploratory study, its pan-cancer screening value requires further validation in prospective multicenter cohorts.	[165]
Ovarian cancer	Small clinical cohort; sample size not reported.	Leave-one-out CV; nine machine learning models; ELISA comparison.	SVM combining labeled and label-free signals: accuracy, sensitivity, and specificity all >95%.	The sample size is relatively small.	[166]
